# Multi-material integrated 3D-printed electrochemical detection platform for rapid on-site screening of nimesulide in industrial sewage

**DOI:** 10.1007/s00604-025-07634-8

**Published:** 2025-10-27

**Authors:** Mateusz Cieślik, Magdalena Rucka, Gilvana P. Siqueira, Adrian Koterwa, Michał Rycewicz, Rodrigo A. A. Muñoz, Robert Bogdanowicz, Jacek Ryl

**Affiliations:** 1 Division of Electrochemistry and Surface Physical Chemistry, Faculty of Applied Physics and Mathematics, Gdańsk University of Technology, Narutowicza 11/12, Gdańsk, 80-233 Poland; 2 Department of Mechanics of Materials and Structures, Faculty of Civil and Environmental Engineering, Narutowicza 11/12, Gdańsk, 80-233 Poland; 3 Institute of Chemistry, Federal University of Uberlândia, Uberlândia, MG 38408-100 Brazil; 4 Department of Analytical Chemistry, University of Gdańsk, Wita Stwosza 63, Gdańsk, 80-308 Poland; 5 Department of Metrology and Optoelectronics, Gdańsk University of Technology, Narutowicza 11/12, Gdańsk, 80-233 Poland

**Keywords:** 3D-printed electrochemical cell, Differential pulse voltammetry, Nimesulide detection, Industrial sewage, Sustainable sensor, Mechano-electric testing

## Abstract

The fabrication, mechano-electric, and electrochemical characterisation of a sustainable, multi-material, integrated 3D-printed electrochemical platform (3DPEC) for the rapid on-site screening of nimesulide in both static and flow conditions is demonstrated. The portable two-electrode cell comprises carbon black (CB)-filled polylactic acid (PLA) electrodes sealed within insulating poly(lactic acid) walls, constituting an integrated multi-material system. Joint tensile and electrical tests, as well as tomography analyses, enabled the optimisation of 3DPEC dimensions and printing parameters. A practical and straightforward route for 3DPEC post-processing activation was proposed to enhance the electrochemically active surface area (ESA). Finally, we present the (sensitive determination of nimesulide, an anti-inflammatory drug, in industrial sewage after a 1:10 (v/v) dilution in phosphate buffer solution (PBS). The detection platform demonstrated high competitive performance in electroanalysis, with excellent reproducibility (RSD = 6.8% by CV, 3.4% by DPV), a recovery of 90 ± 5%, a detection limit of 0.19 μM, and selectivity against most studied interferents. The presented approach serves as a proof-of-concept for a customisable, multi-material integrated 3D-printed electrochemical detection platform that prioritises reproducibility, low cost, and applicability in complex environmental matrices, highlighting its practical potential for sustainable wastewater monitoring.

**Graphical abstract:**

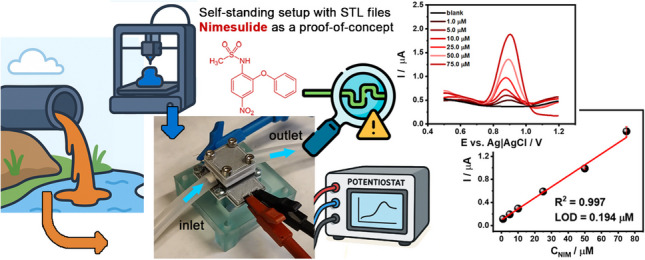

**Supplementary information:**

The online version contains supplementary material available at 10.1007/s00604-025-07634-8.

## Introduction

Non-steroidal anti-inflammatory drugs (NSAIDs) such as ibuprofen, diclofenac, or nimesulide (NIM) are commonly consumed pharmaceuticals. Their widespread use has led to increasing environmental concerns, primarily because many of these drugs are excreted unmetabolised, entering wastewater treatment systems. Their effective removal is challenging, as they pose potential ecological and health hazards due to their persistence and bioactive properties, even at low concentrations. Compared to other NSAIDs, NIM is known to have potentially toxic effects on aquatic organisms, influencing their development and reproductive health [1, 2]. This emerging contaminant poses a threat to water ecosystems by disrupting microbial communities, promoting antibiotic resistance, and negatively impacting aquatic organisms [3, 4].

**Fig. 1:**
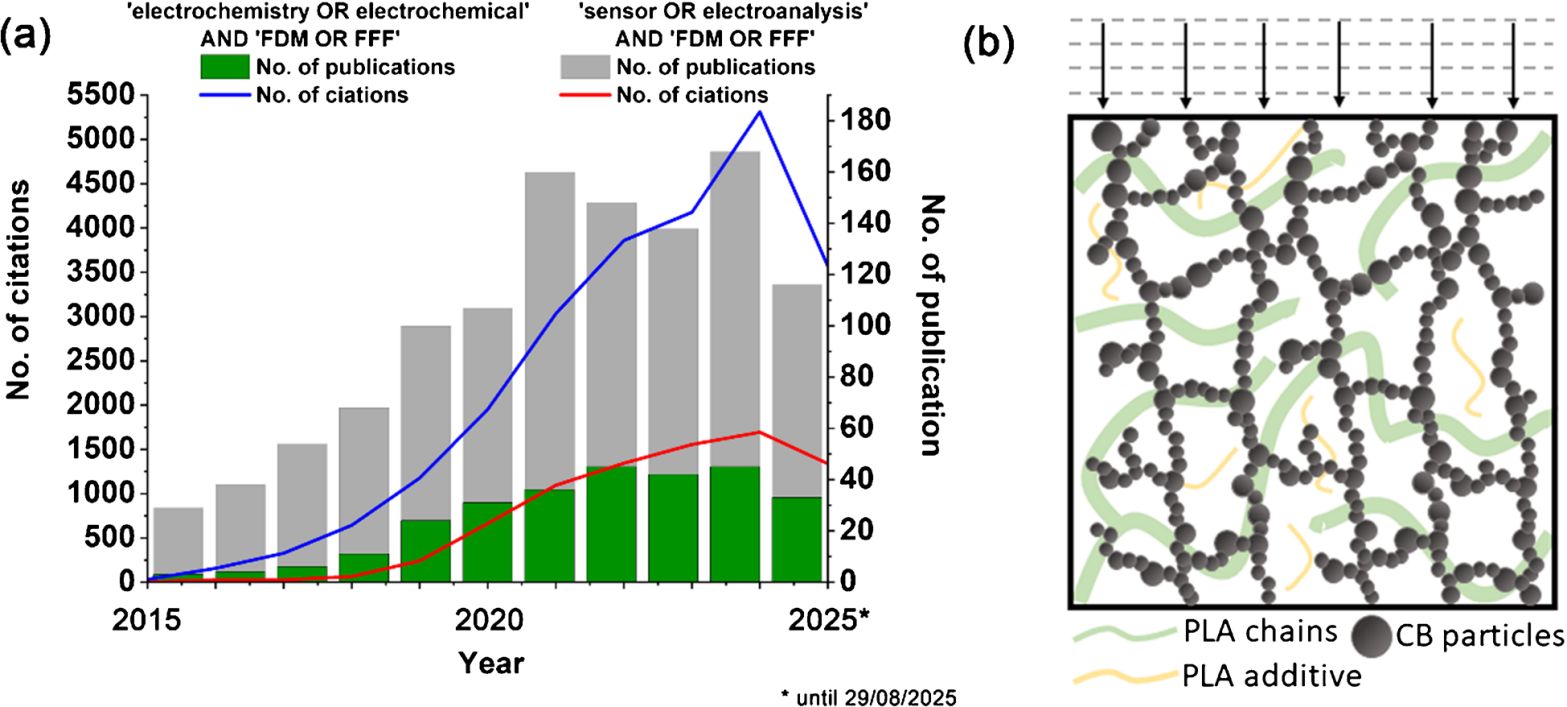
**a** Growth of attention to 3DP electrochemical sensors in JCR articles (source: Scopus database, 29/08/2025); **b** extrinsic conductivity by carbon nanoparticles in 3DP fillers, offering heavily overlapping diffusion field distribution around uncovered conductive filler particles after surface activation

Compared to traditional production methods, 3D printing (3DP) by material extrusion offers several advantages, including fast and flexible design, the possibility of prototyping, mass customisation, innovation in business models, and environmental friendliness through waste reduction and recycling [5–7]. One of the most promising applications of 3DP in electrochemistry concerns sensing [8, 9], where the number of reports and citations is growing exponentially, as shown in Fig. 1a. 3D-printable sensors offer a promising approach for rapid, cost-efficient, and customisable monitoring of chemicals in environmental and biological matrices. They thus could play a crucial role in addressing concerns related to potential health and ecological impacts by enabling rapid on-site analysis and early intervention. 3DP electrodes have been used to detect trinitrotoluene [8], tetracycline [9], dopamine [10], and caffeine [11], among other substances. However, only a few available reports focus on pharmaceutical diagnostics [12, 13], usually reaching micromolar detection limits.

Most available reports focus on the use of commercially available filament dedicated to 3D printing, ProtoPasta®, which is fabricated from thermoplastic polymer poly(lactic acid) (PLA) and carbon black (CB) filler. Many authors have fabricated dedicated composites with different fillers, i.e. carbon black (CB) [14], carbon nanotubes [15], graphene [16], or even nanodiamond derivatives [10] in an amount sufficient to fabricate percolation conductive paths (see Fig. 1b). Such a procedure makes a 3D printout an attractive electrode material. Moreover, 3DP fits perfectly into the circular economy and integrates emerging Industry 4.0 technologies [17]. This will enable moving away from the concept of waste and the linear ‘take-make-waste’ model, replacing it with the 6R model based on the principles of reduce, reuse, recycle, recover, redesign, and remanufacture [18, 19].

Since material extrusion is one of the few technologies that support multi-material 3DP, combining conductive and insulating materials into more complex architectures makes it possible to build electronic components [20], electrochemical cells [21], and other devices. A three-electrode 3D-printed electrochemical cell (3DPEC) was presented in the work [22], where it was used to detect cadmium, lead, uric acid, and midazolam maleate ions. Kokkinos and his research team created dual-extruder printed systems in which the measurement cell was printed from a conductive PLA composite [23] and from an ABS composite [24, 25]. Both devices were used to analyse pharmaceutical and environmental samples. Measuring cells with electrodes produced by other authors in one printing process were used for the detection of dopamine [26], ascorbic acid [27], hydrazine oxidation [14], or ephedrine detection [15]. Li et al.’s [16] freedom to design the shape led to the fabrication of a fully functional 3DP flow cell, used to analyse pharmaceuticals in biological fluids, and produced results as good as those of conventionally used systems.

An important yet often overlooked aspect of 3D printing (3DP) is internal integrity, which originates from the mechanical strength of the manufactured components, particularly multi-material joints. Carbon fillers change the strength parameters of prints, most notably increasing the hardness [28] and improving the wear resistance [29] of printed components. Many studies have focused on optimising the printing parameters, such as deposition speed, nozzle temperature, bed temperature, layer thickness, infill density, and infill pattern [30–32]. Finally, the electric properties depend, to a large extent, on the 3DP operating temperature or mechanical deformation [33], which may effectively translate into issues with sensor stability or reproducibility. Reports showcasing joint mechano-electric characteristics of conductive and non-conductive complex structures fabricated in a single 3D printing process are missing.

This work proposes an integrated, optimised, and easy-to-print multi-material 3D-printed electrode array, verified as 3DPEC for electroanalytical studies in both stationary and flow-through conditions. The cell comprises a two-electrode setup built from CB-filled, electrically conductive PLA and enclosed in insulating PLA. The manuscript explores the possible influence of 3D print geometry and structure on reproducibility, trying to understand the role of tensile stresses on electric properties, multi-material interface integrity, and intermaterial CB diffusion effect on electrochemically active surface area (ESA) reproducibility. We have presented an in-depth electroanalytical study of such 3DPEC, using the example of a prone-to-fouling analyte, nimesulide. The complex industrial sewage matrix enabled the definition of the actual value of the developed 3DPEC in terms of reproducibility, selectivity, and detection limits. In this work, the term industrial sewage refers to treated wastewater effluents from industrial facilities. Such chemically complex matrices are particularly challenging for electrochemical detection due to fouling effects and overlapping redox processes, yet they represent a realistic target environment for practical monitoring applications [34].

We fabricated the devices using a commercial filament, and the STL files of the developed 3DPEC were published and made available for unrestricted use. Overall, this report serves as a proof of concept for a cost-effective 3DPEC for rapid screening in real-world environments.

## Experiment

### Materials for 3DPEC and reagents

All the samples were manufactured using a Prusa i3 MK3S + with an MMU2S multi-material module (Prusa Research, Czech Republic). The 3D printing parameters applied for PLA (Raise3D, Irvine, USA) and CB-PLA (Protopasta Inc, Vancouver, USA) were as follows: nozzle diameter, 0.4 mm; nozzle temperature, 220 °C; building platform temperature, 60 °C; layer thickness, 0.2 mm; infill, 40%; first six and last six layer infill, 100%; extrusion multiplier, 1.1; print speed, 40 mm/s; first layer print speed, 20 mm/s; and the infill lines were printed at + 45°/− 45° angles. We used higher printing temperatures to affect the mechanical and electrical properties, resulting in increased fusion between the layers [33, 35]. This is a beneficial observation from the perspective of printing composite materials with carbon fillers, as printing these materials at too low a temperature can lead to clogging of the nozzle. The infill pattern has only a slight effect on the mechanical properties [36]. The 40% infill was proposed to optimise the material use and printout duration, resulting in significant material and energy savings. One hundred percent infill was only used for the six layers at the bottom and top of the electrode 3DP, with conductive material, to ensure leak-tightness of the print during measurements in water solutions, as visible in the cross-section of the electrode (Fig. S2). For comparison, the printout of the sample used in the mechanical tests, using the proposed parameters, took 49 min and consumed 11.2 g of PLA. Printing the same sample with 100% infill takes 59 min (20% longer duration), and the sample weight is 14.5 g (29% higher consumption). We chose the CB-PLA/PLA interface due to similar printing parameters of both composites, good adhesion, and biodegradability. The use of other materials is limited by the lack of availability of conductive composites, poor adhesion to PLA (e.g. PET-G), or mismatched printing temperatures with PLA (e.g. ABS). All the files necessary to 3DP the flow measurement electrochemical cell, electrodes, and top lid can be downloaded from the link in the Reference section [36]. The above STL is available for unrestricted use under the CC BY 4.0 licence, and the authors kindly request that a reference be included for this manuscript. The 3D printing and performance of the 3DPEC are visualised in a video attached to the Supplementary Information (SI) file.

Redox probes, K_3_[Fe(CN)_6_] and K_4_[Fe(CN)_6_], and phosphate-buffered saline (PBS), pH 7.4, concentration 0.01 M, were purchased from Sigma-Aldrich. Nimesulide was obtained from Alfa Aesar (Thermo Fisher Scientific). Caffeine, paracetamol, and diclofenac sodium were purchased from Sigma-Aldrich. Resorcinol was obtained from Acros Organics. Ciprofloxacin hydrochloride monohydrate was acquired from Tokyo Chemical Industry Co., Ltd.. Ascorbic acid was purchased from Fisher Chemical. All reagents were analytical grade. The conductivity of the distilled water was *k* = 0.05 µS, and the pH = 7.94. A sample of treated sewage was collected from the treatment plant in Dębogórze (Pomerania, Poland). Based on the high-performance liquid chromatography experiment, no NIM was present in the sewage. The composition and basic physicochemical parameters are presented in the SI file, Table S1.

### The mechano-electric characteristics of multi-material 3D-printed samples

The tensile resistance studies were performed on dog-bone-shaped samples with a length of 150 mm and a cross-section of 15 × 4 mm^2^. Different variants were prepared, including homogeneous, notched, and multi-material samples of various geometries, as depicted in the SI file, section S2. The experiment used a Zwick/Roell Z10 universal testing machine (ZwickRoell GmbH & Co. KG, Ulm, Germany). The samples were loaded until failure at a constant displacement rate of 1 mm/min. During the tensile test, the mechanical behaviour of the samples was characterised using the digital image correlation (DIC) method. Images of the front surface of the samples were taken every 1 s using an ARAMIS MC 3D 12 M system (GOM GmbH, Braunschweig, Germany). The collected images were then processed using the Aramis Professional software (GOM GmbH, Braunschweig, Germany) to analyse sample deformation. The setup is shown in the SI file, Fig. S3.

**Fig. 2:**
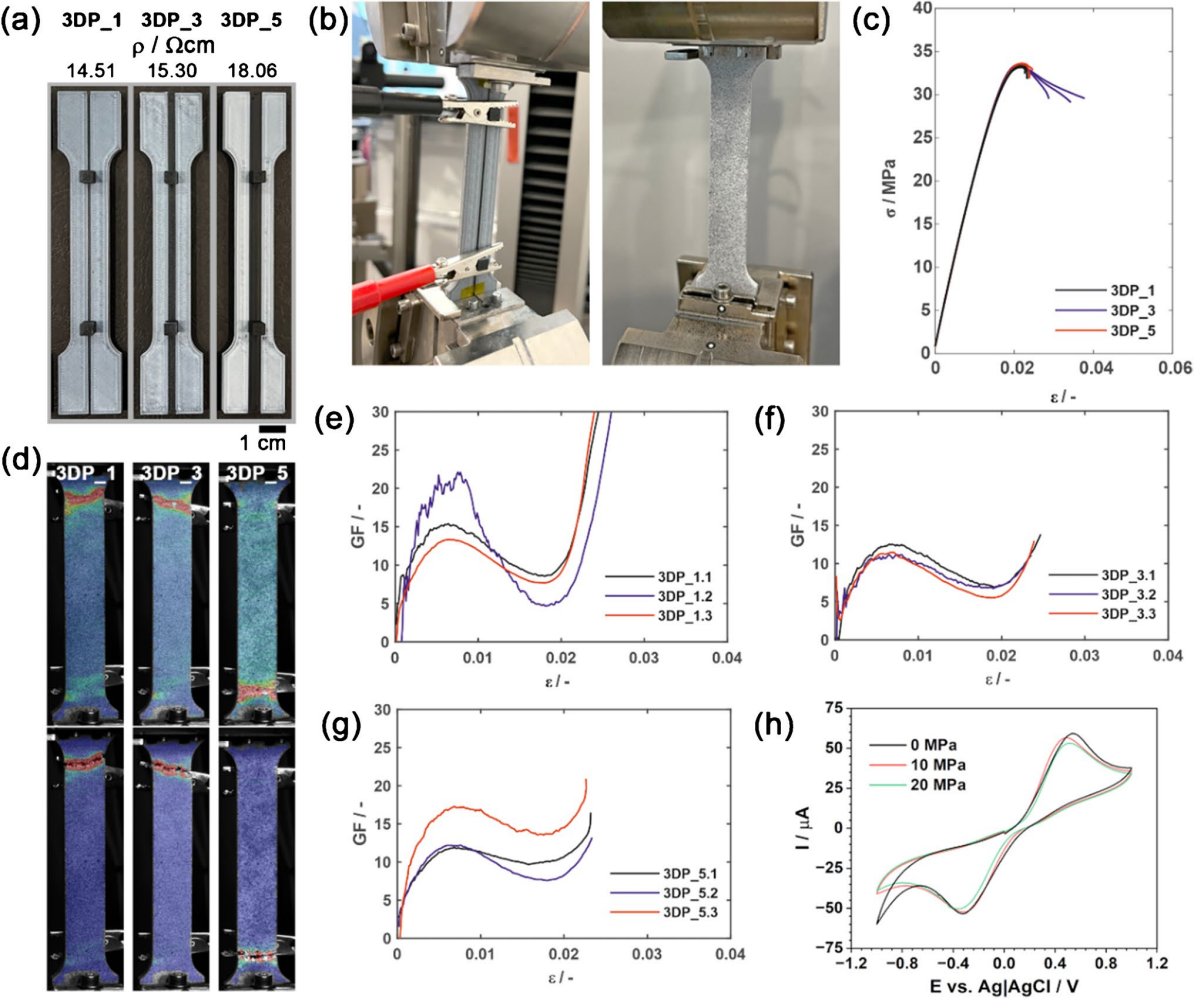
** a** 3DP samples and resistivity ρ: strap sizes *N*×2×50 mm^3^ where *N* 1, 3, or 5 mm (labelled as 3DP_N); **b** sample fixed for mechano-electric tests, painted for Mises strain maps; **c** stress-strain curves of three 3DP_1 samples (1 mm strap thickness); **d** DIC analysis results in the form of Mises strain maps before failure (first row) and after failure (second row); **e**–**g** GF of **e** 3DP_1; **f** 3DP_3 and **g** 3DP_5 as a function of applied strain *ε*; **h** CV (100 mV/s scan rate) for 3DP_3 sample at rest and after elongation at 10 MPa and 20 MPa. The scan was performed from 0.0 to +1.0 V; +1.0 to −1.0 V; −1.0 to 0.0 V

Samples depicted in Fig. 2a had electrically conductive CB-PLA pathways, with dimensions N × 2 × 50 mm^3^, where *N* = 1, 3, or 5 mm, to measure changes in electrical properties during the tensile resistance tests. These samples were labelled as 3DP_N. For this purpose, electrodes with printed contacts (as seen in Fig. 2b) are attached to a source-measure unit (2450, Keithley). The distance between the electrodes was 70 mm. Resistance was measured in a two-wire configuration, with a constant current value of 100 μA set for all samples.

### Electrochemical measurements

The electrochemical measurements were conducted using an Autolab PGSTAT302N potentiostat/galvanostat (Metrohm Autolab, Utrecht, Netherlands), with data acquisition and processing performed using NOVA 2.1.8 software. The measurements were performed on all three 3D-printed two-electrode (working electrode, WE, and counter electrode, CE) 3DPECs, as shown in Figs. 4c and 5a, enclosed in a flow cell. An embedded silver rod served as the reference electrode (RE). Loading CB-PLA with Ag nanoparticles could offer a viable alternative for pseudo-RE [37, 38], yet such composites are not yet commercially available. Moreover, printed RE electrodes face numerous challenges related to their instability and susceptibility to contamination, which limits their use in systems requiring robustness [39]. The geometric surface area of each WE and CE was 0.84 cm^2^, and the volume of the 3DPEC flow cell was approximately 1.6 cm^3^. An activation procedure was carried out by electrochemical saponification in 1 M NaOH during ten polarisation cycles at a constant scan rate of 100 mV/s, from − 1.4 to + 1.2 V [40].

The 3DP electrode kinetics were studied using cyclic voltammetry (CV) and electrochemical impedance spectroscopy (EIS) using 0.01 M PBS with 1 mM [Fe(CN)_6_]^3−/4−^. The scan rates used in CV studies ranged from 10 to 100 mV/s. The EIS analysis was performed under open circuit potential (OCP) in the frequency range from 100,000 to 0.1 Hz, with 10 points per frequency decade and an amplitude of 10 mV. ESA estimation was based on the modified Randles–Sevcik equation for an irreversible, one-step, one-electron reaction [41], as shown in Eq. (1):

1 $${i}_{\left(\text{a},\text{p}\right)}=2.99\bullet {10}^{5}{\alpha }^{\left(1/2\right)}A{C}_{O}{D}_{O}^{\left(1/2\right)}{\upsilon }^{\left(1/2\right)}$$

where *ν* is the scan rate (V/s), *α* is the charge transfer coefficient (set to 0.5), *A* is the ESA in cm^2^, and *D*_0_ is the diffusion coefficient, which for [Fe(CN)_6_]^3−/4−^ is approx. 6.67 × 10^−6^ cm^2^/s [42], and *C*_0_ is the concentration of the electrochemically active compound (mol cm^−3^).

Differential pulse voltammetry (DPV) was used for NIM determination. The DPV measurements were performed under static conditions, after the electrolyte was pumped into the cell using a peristaltic pump (model PP 1–05, Zalimp, Poland). After completion of the measurement, the PBS was pumped again to clean the 3DPEC (50 mL/min for 5 min) before the NIM concentration was increased for the next test. DPV was operated under the following conditions: modulation amplitude, 50 mV; modulation time, 7 ms; interval time, 1.85 s; and step potential, 0.01 V, all of which were selected to ensure adequate peak resolution, stable signals, a low signal-to-noise ratio, and a scan rate of 5 mV/s. The NIM was detected in a potential range of + 0.5 to + 1.0 V in 0.01 M PBS (pH = 7.01) and in treated industrial sewage (pH = 6.92) [43, 44]. PBS was chosen for the electrochemical analyses because it is one of the most widely used buffers in environmental studies involving complex samples [45, 46]. The selection of PBS ensures a better representation of the experimental conditions and a pH value close to the NIM pKa (6.56 [47]), where the molecule is highly active [48]. The NIM peak current values presented were processed using NOVA 2.1.8 software, which calculates the maximum peak height by drawing a baseline from the start to the end of the peak. The NIM limit of detection (LOD) was estimated using Eq. (2).

2 $$LOD=\frac{3 \times STD}{m}$$

where *STD* represents the standard deviation obtained from ten blank signal measurements obtained by DPV (STD = 1.239 × 10^−9^), and *m* denotes the slope of the calibration curve.

The applicability of the proposed method was evaluated using industrial sewage samples collected at the Dębogórze treatment plant (Pomerania, Poland). The samples were stored in amber bottles under refrigeration. Before each experiment, they were diluted in a supporting electrolyte at a 1:10 (v/v) ratio (0.01 M PBS, pH 7.01: sewage). The diluted samples were then introduced into the electrochemical cell for immediate measurement, both in the presence and absence of NIM.

### Physicochemical studies

A 3D X-ray microscope (XRM) combines micro-computed tomography with microscopic visualisation. These analyses were conducted using a Bruker Skyscan 2214 CMOS 3D X-ray microscope, operating a nanofocus X-ray source with a focal spot below 500 nm. The voxel size was 1.6 μm, and the operating voltage was 45 kV. The 3D.SUITE software was used for visualisation. These studies were performed on small, 10 × 10 × 4 mm^2^ printouts with 100% infill. X-ray photoelectron spectroscopy (XPS) analyses were performed using an Escalab 250Xi spectroscope (Thermo Fisher Scientific). The spectroscope operates an AlKα x-ray source (spot size 250 μm). The pass energy was set at 20 eV. Low-energy electron and Ar^+^ ion bombardment was used for charge compensation, with a final peak calibration using adventitious carbon C1s (284.6 eV). Avantage v5.9921 software was used for peak deconvolution.

The contact angle measurements were conducted using a DSA100 drop shape analyser (Krüss, Germany). A drop of 2 μL of standard liquid (water) was placed using a syringe at room temperature, and a CCD camera connected to the graphics card recorded the image of the drop. Twenty repetitions of the measurements were made. The average contact angle was determined after digital image analysis using the Young–Laplace method.

## Results and discussion

### Mechano-electric properties of multi-material samplesMechano-electric properties of multi-material samples

Multi-material 3D printing introduces significant complexity regarding the mechano-electric properties of the studied composites. The applied stresses affect elongation and the subsequent breaking of conductive paths [49], which can significantly impact composite resistance and, consequently, the electrochemical performance [33]. This effect is further dependent on printing orientation. CB-filled composites reveal lower tensile resistance than their non-conductive counterparts; a multi-material joint is expected to be characterised by more complex behaviour [50]. Moreover, diffusion of the carbon black filler from the CB-PLA into the insulating PLA at the joint may form excessive conductive paths and uncontrollably modify the electrode’s ESA.

To verify 3DPEC susceptibility to the above-described factors, a series of tensile tests with superimposed electric resistance measurements was performed. Notably, carbon black addition to polylactic acid has the effect of reducing the average tensile strength from 33.81 to 20.24 MPa (see Fig. S4), while calculated elastic modulus and Poisson’s ratio indicate lower stiffness of the CB-PLA samples compared with the pure PLA, which are as follows: *E* = 2.23 GPa, *v* = 0.33 (PLA), and *E* = 1.63 GPa, *v* = 0.35 (CB-PLA). Introducing multi-material joints in the form of CB-PLA stripes results in a reduction of tensile strength due to stress concentration zones. The distribution of Mises strains for the samples just before and after failure (SI file, Fig. S4) reveals that the PLA failure occurs due to exceeding the maximum normal stresses, as characteristic of a material with predominantly brittle properties. For multi-material samples, the nature of the damage was similar to that of single-material samples, as the elastic moduli of the PLA and CB-PLA are not significantly different. It should be concluded that for multi-material samples, the presence of CB-PLA impacts the structure negligibly, as the cross-sectional area of the conductive strap is small.

The GF parameter describes the ratio of relative change in electrical resistance FRC with strain ε (explained in detail in the SI file, section S3 with auxiliary mechano-electric tests). All studied samples exhibit an increase in GF with elongation, most notable for the thinnest, 1 mm CB-PLA stripe, as shown in Fig. 2d (SI File, Fig. S6 for other samples). Here, alternative charge flow paths could not occur, as with wider samples, even leading to a lack of conductivity for one of the samples when prominent tensile stress was applied. The electrical resistance increases nearly instantly after applying the tensile stress, suggesting that small stresses may lead to several percentage changes in ΔR/R. At higher stresses, a GF = f(ε) plateau is noted and connected with the CB-PLA material yielding. Pure, insulating PLA exhibits a higher degree of crystallisation, resulting in a higher flexural strength of the samples compared to CB-PLA composites. As a result, it remains in the elastic region as the primary phase, while CB-PLA deforms plastically at lower stress levels. Resistance measurement refers only to conductive paths and shows CB-PLA composite plasticisation, while the stress–strain curve of the whole sample, predominantly composed of PLA, remains in its *quasi*-elastic range. Given the mechano-electric analyses, the 3DPEC electrode connectors in the design should be at least 3 mm thick.

Most importantly, changes in resistance resulting from the tensile test in the sample elastic range do not affect the electrochemical response, which was verified by repeating the voltammetry study of [Fe(CN)_6_]^4−/3−^ redox process, see Fig. 2 h at rest and after elongation at 10 and 20 MPa, which is by far surpassing the expected mechanical stresses induced during 3DPEC operation. The CV peak currents only slightly diminished under the applied load, indicating a loss of some conductive paths within the material and an increase in electric resistance. Yet, this change does not surpass 6% even under the highest applied loads and is within the limits of statistical error for similar tests at rest. Notably, the experiment did not lead to an observable increase in the peak-to-peak separation Δ*E*, which, if it occurred, would imply an increase in surface electric heterogeneity upon conductive path loss [51].

**Fig. 3:**
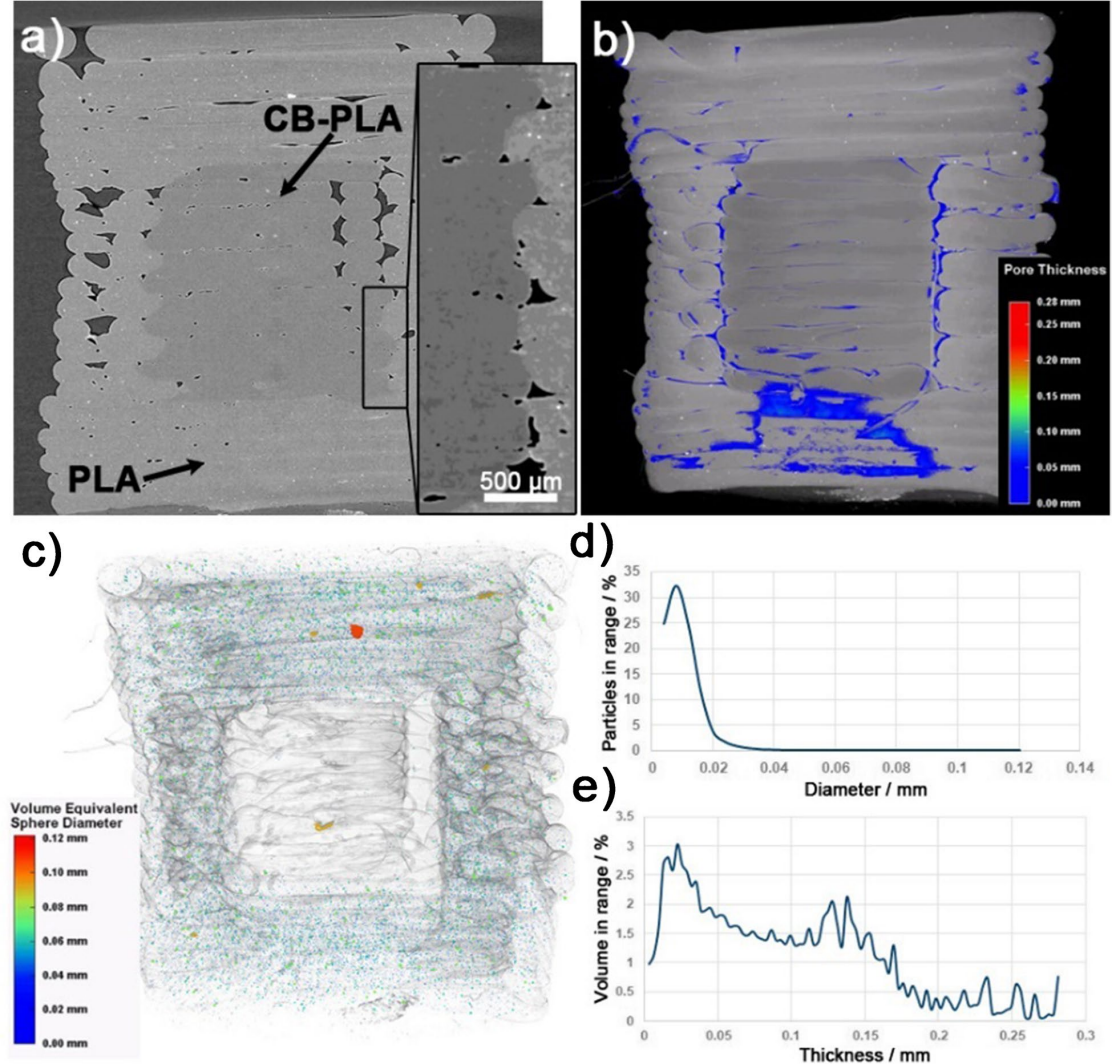
**a** XRM micrography of the studied sample; **b** 3D pore thickness distribution; **c** 3D particle size analysis; **d** particle size distribution, and **e** pore thickness distribution

The CB-PLA/PLA interface is of particular importance, considering the mechanical properties of the final printout, the leak-tightness, and the electrochemically active surface area of the electrochemical cell. The XRM micrograph in Fig. 3a reveals a difference in composite density between the two materials, with lower-density particles observed for the conductive CB-PLA (visualised darker). The inset figure created with a Phase Retrieval Data tool provides a better visualisation of the boundary between the two phases and a compact connection with only a few pores at the interface. The presented visualisation makes it reasonable to conclude the presence of a sharp interface and negligible CB diffusion into the insulating PLA. Based on previous findings [10], the CB volume sufficient to form percolation paths is approximately 15%. Thus, one should expect that the electrode ESA is unaffected by joint formation.

Pores appear naturally, even with 100% infill, both at the multi-material interface and between the printed layers and may lead to small geometric surface and ESA variations between 3DP samples. These are visualised with colours in Fig. 3c and the porosity volume distribution in Fig. 3e, testifying that the pore thickness does not exceed 150 μm and the pore volume is 5.32%. Notably, one must consider that this value is highly dependent on the 3DP conditions and the printer model. The analysis of the particle distribution within the 3DP sample, depicted in Fig. 3d, reveals the filler with a sphere diameter in the range of 10 and 120 μm. A higher volume equivalent sphere diameter is observed for the insulating PLA sample compared to the CB-PLA, most likely related to the pigment and other fillers used by the manufacturer. The CB filler, which falls in the nanometre range, could not be observed. This analysis further confirms the absence of CB aggregates within the material that may occur during filament processing, which significantly deteriorates the electrical and electrochemical characteristics [49].

### Electrochemical characteristics of the 3DPECElectrochemical characteristics of the 3DPEC

The utilisation of 3DP electrodes as electrochemical sensors is most often preceded by the so-called activation procedure, which aims to remove the top polymer layer and expose the conductive filler to the electrolyte, boosting the charge transfer kinetics [7]. The activation may be achieved by different means, such as [52] polishing, hydrolysis in aprotic solvents [53, 54], or reducing agents [55], enzymatic digestion [40, 56], electrochemical saponification [26, 40, 57], plasma [58], laser ablation [59, 60], or even a kitchen microwave oven [61]. Some of these approaches would be difficult to execute within a complex-shaped object.

**Fig. 4:**
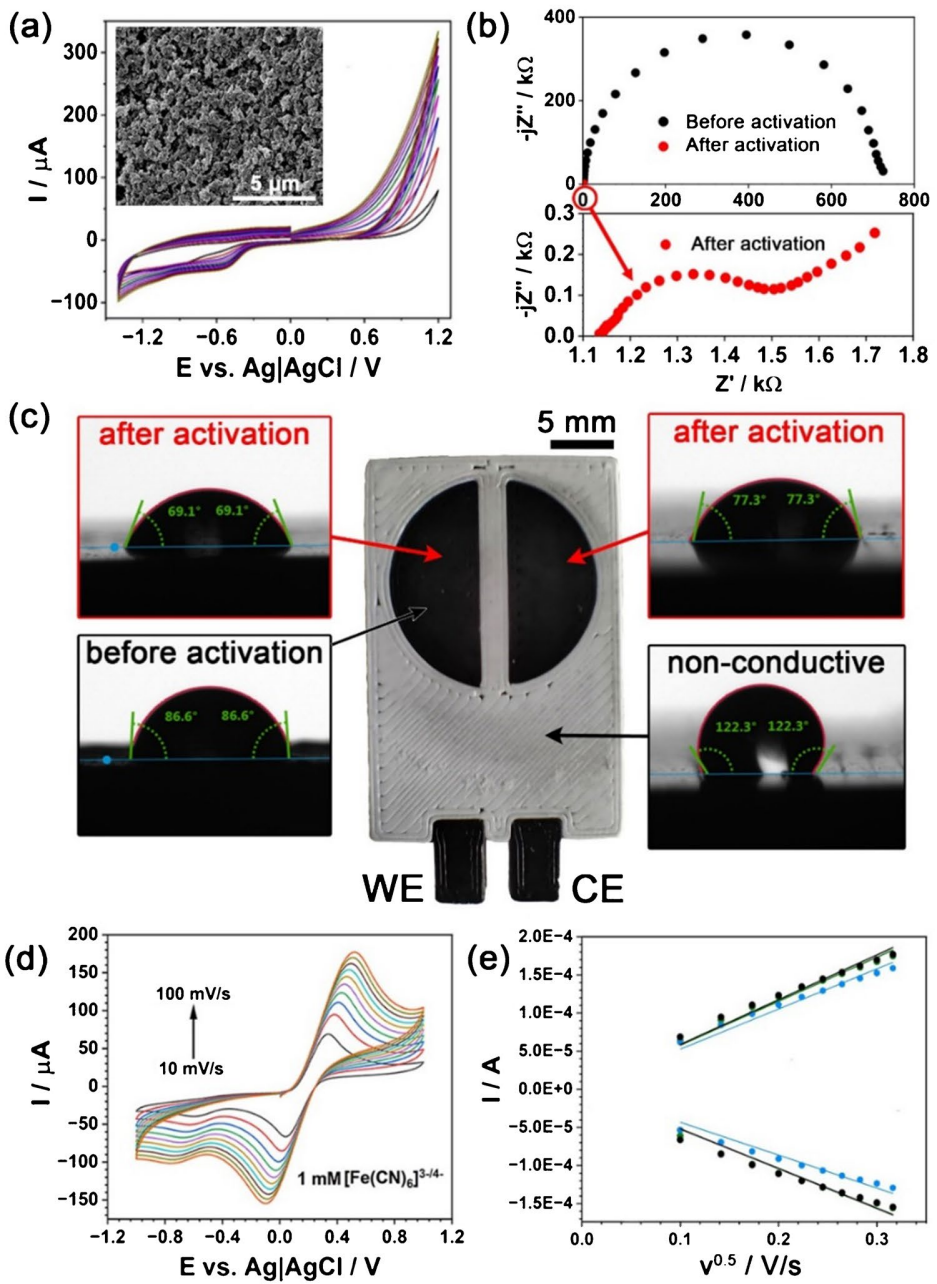
**a** CV curves registered during sample activation with SEM micrograph after activation in the inset. The scan was performed from 0.0 to +1.2 V; +1.2 to −1.4 V; −1.4 to 0.0 V; **b** Nyquist plots obtained (1.0 mM [Fe(CN)_6_]^4−/3−^ + 0.01 M PBS, at OCP conditions) before and after activation; **c** photograph of a free-standing 3DPEC with contact angle measured for both PLA and CB-PLA prior to and after activation; **d** CV at different scan rates for 3DPEC in 1.0 mM [Fe(CN)_6_]^4−/3−^ + 0.01 M PBS, and the scan was performed from 0.0 to +1.0 V; +1.0 to −1.0 V; −1.0 to 0.0 V; **e** peak current vs scan rate square root for three independent 3DPECs

Considering our aim to build a closed 3DPEC operating under flow conditions, electrochemical saponification stands out as a versatile option, where PLA removal is only observed for the conductive CB-PLA composite. The CV scans registered during the 3DPEC activation are presented in Fig. 4a. The figure inset illustrates electrode topography developed under the activation protocol. The efficacy of the 3DPEC activation was confirmed by EIS analyses (Fig. 4b) and CV (SI file, Fig. S7) in the presence of the [Fe(CN)_6_]^4−/3−^ redox probe. The value of charge transfer resistance (diameter of the semicircle in the Nyquist plot) decreases by over two orders of magnitude, from ~ 720 to merely 0.4 kΩ (see SI file, Table S4).

A photograph of a non-enclosed 3DPEC version is presented in Fig. 4c. This 3DPEC version was used for contact angle studies, again showing the superior adaptability of 3DP measurement setups. The CB-PLA (before activation, 86.60° ± 4.24°) and non-conductive PLA (122.16° ± 0.91°) differ significantly in the average contact angle value, allowing us to conclude the CB’s role in increasing composite hydrophilicity. Following this observation, contact angle measurement is a valid tool for studying the efficacy of activation. The activation protocol further decreases the contact angle, as measured for both WE (69.52° ± 1.56°) and CE (76.99° ± 1.31°), indicating the influence of the activation process on the physical properties of both electrodes. A lower contact angle value indicates better wettability, which should benefit applications that require interaction with liquids. Good pore wetting contributes to an ESA increase. This effect is primarily connected to PLA removal from the surface and the formation of macropores, introducing capillary effects and a thoroughly wetted Wenzel model state [62]. On the other hand, activation may influence CB surface oxidation, producing carboxyl and hydroxyl surface functional groups [40]. Overall, the study confirms that the proposed activation protocol, as determined by CV scans in a wide polarisation range (visualised in the SI file, Fig. S8), offers a unique feature to activate both WE and CE simultaneously. Auxiliary tests were performed with the use of pseudo-RE (3D-printed and Ag ink–coated); see SI file, Fig. S10 and S14. Such systems reveal potential shifts of up to 200 mV and higher capacitive currents, where the presence of binders leads to heterogeneous electrode behaviour. Furthermore, in flow systems, instability is increased by silver leaching and fouling by contaminants on porous surfaces.

Next, CV curves at different scan rates were registered to estimate the ESA of the WE within the 3DPEC, as seen in Fig. 4d. This experiment revealed high, well-developed peak currents with negligible impact from non-Faradaic phenomena. It should be noted that the i_P,C_ is considerably lower than the i_P,A_, suggesting a hindered reduction process and reaction irreversibility. Simultaneously, the skewed peak shape and notable peak-to-peak separation Δ*E* are caused by the ohmic drop iR due to the use of CB-PLA as both WE and CE [63]. The series resistance within the 3DPEC exceeds 1.1 kΩ, as measured by EIS (Fig. 4b). While there are means to compensate for the iR drop, its effect largely depends on the pre-assumptions regarding the studied system [64]. This observation also highlights a key consideration for future designs, such as reducing the WE-CE distance or connector length. Many studies aim to develop filaments with higher conductivity to enhance the reversibility of the redox process [65, 66].

Peak currents i_P_ vs. CV scan rate square root $\sqrt{\nu }$ depicted in Fig. 4e for three independent samples demonstrate a linear behaviour, characteristic of the Randles–Sevcik formula and its approximation for irreversible redox processes [67]. Following the observed reaction irreversibility, the ESA estimated for [Fe(CN)_6_]^4−^ oxidation was 12% higher compared to [Fe(CN)_6_]^3−^ reduction, reaching 0.89 ± 0.05 cm^2^ and 0.78 ± 0.08 cm^2^, respectively. Thus, the value of ESA reflects the WE geometric surface area (0.84 cm^2^), in a nearly 1:1 proportion. The reproducibility of the [Fe(CN)_6_]^4−/3−^ kinetic studies and the calculated ESA should be considered satisfactory, particularly in light of the effects of the inner-sphere electron transfer mechanism of the redox probe, the iR drop, and unavoidable geometric imperfections of the printout. The low variation in the ESA results confirms the XRM studies regarding the low material mixing at the CB-PLA/PLA interface.

### Nimesulide detection using 3DPEC in the industrial sewage

**Fig. 5:**
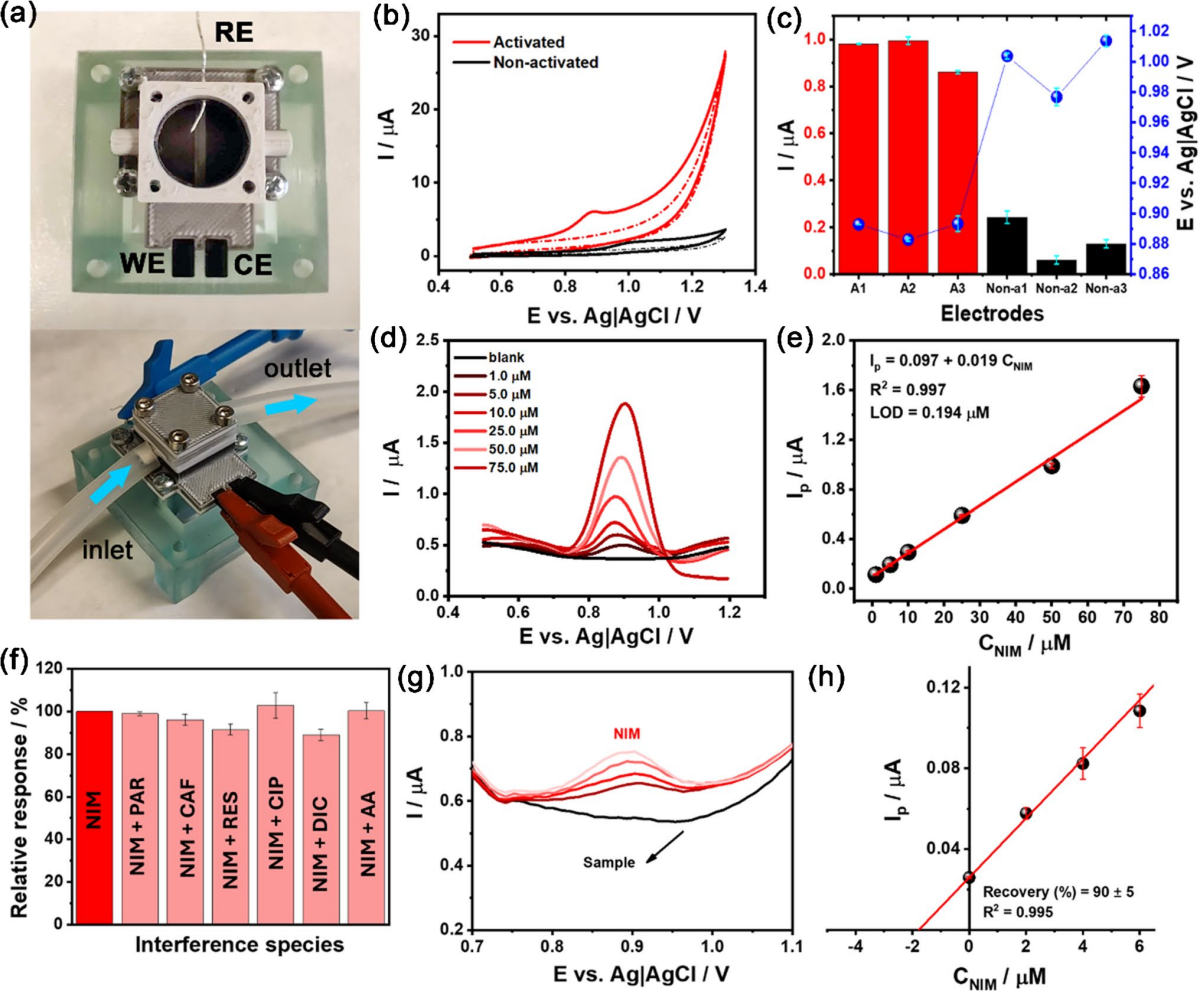
**a** Photograph of a free-standing flow-through 3DPEC; **b** CV responses of 0.2 mM NIM using 3DP electrodes was performed at activated (red line) and non-activated (black line) electrodes, with scan rate of 100 mV/s. Nimesulide was dissolved in a diluted mixture of 1:10 (v/v) (0.01 M PBS, pH 7.01: sewage). The dashed lines represent the corresponding blank signals (0.01 M PBS, pH 7.01: sewage). The scan was performed from +0.5 to +1.3 V; +1.3 to +0.5 V; **c** corresponding variation in current intensities and peak potentials from CV measurements of three successive scans (*n* = 3) on three activated and three non-activated electrodes in the presence of NIM; **d** DPV analysis with 3DPEC at different NIM concentrations (1.0 to 75.0 μM NIM) in industrial sewage. NIM was dissolved in a diluted mixture of 1:10 (v/v) (0.01 M PBS, pH 7.01 : sewage); **e** respective calibration curve. The error bars in Fig. 5e represent the standard deviation derived from measurements (*n*=3) at the same concentration; **f** relative variation (*n*=3) of each interfering species in the electrochemical response of NIM, using an interfering agent/NIM ratio of 1:1, at a concentration of 10.0 µM; **g** DPV measurements were carried out using the activated electrode for the analysis of a sewage water sample (black lines). The sample was initially spiked with 2.0 μM NIM (dark red line), followed by successive additions of 2.0 μM NIM (light red lines); **h** the corresponding calibration curve (*n*=3) for NIM was constructed based on the standard additions. Nimesulide was dissolved in a diluted mixture of 1:10 (v/v) (0.01 M PBS, pH 7.01: sewage). The error bars in Fig. 5h represent the standard deviation derived from the measurements (*n* = 3) for each added concentration

The 3DPECs were studied for electrochemical NIM detection in industrial sewage, employing DPV. These studies were performed on 3DPEC with identical geometry as presented in the ‘Electrochemical characteristics of the 3DPEC’ section, but with a closed flow-through cell (volume 1.6 cm^3^) overprinted at the top of the electrodes; see Fig. 5a. The flow-through 3DPEC allows adequate mixing and 1:10 (v/v) dilution (0.01 M PBS, pH 7.01: sewage).

The 3DPEC enabled us to conduct both qualitative and quantitative identification of NIM. Figure 5b displays the cyclic voltammetric NIM response at both activated and non-activated electrodes. The dashed lines indicate the absence of peaks in 0.01 M PBS, pH 7.01: sewage water. Upon the addition of 0.2 mM NIM, a single irreversible oxidation peak [68] emerged at approximately 0.9 V for the activated electrode and around 1.0 V for the unactivated one. The anodic signal originates from the oxidation of the methylsulfonamide group. Notably, the peak current at the activated electrode was 16 times higher. This effect may be attributed to an increased electroactive surface area resulting from activation and/or enhanced catalytic activity, consistent with previous observations [69]. The repeatability of NIM detection was assessed using three electrodes, both activated and non-activated, as shown in the SI file, Fig. S11a,b. A comparison of the NIM peak currents and oxidation potentials for both electrode types is presented in Fig. 5c. Notably, a relative standard deviation (RSD) of 6.8% in peak currents was observed among the activated electrodes, whereas the non-activated electrodes exhibited a much higher RSD of 56.4%. This effect suggests that activation improves both the sensitivity and the reproducibility of NIM detection on activated surfaces. The non-activated electrodes exhibit greater signal variability, likely due to surface fouling, discussed later. A similar effect is observed in the peak potentials, with a variation of just 0.6% for the activated electrodes, compared to 1.7% for the non-activated ones.

Then, the DPV analyses were performed to estimate the LOD (Fig. 5d). The resulting calibration curve (1.0 to 75.0 μM NIM; *R*^2^ = 0.997) (Fig. 5e) reveals a linear function spanning the micromolar NIM concentration (*I*_p_ (μA) = 0.0972 ± 0.0043 + 0.0191 ± 0.0006 *C*_NIM_) range with an estimated LOD value of 0.194 μM. When comparing the analytical parameters of the proposed method with those obtained using a conventional electrode, such as glassy carbon (SI file, Fig. S12), under identical conditions (pH, supporting electrolyte, sample, DPV parameters, and NIM concentration), a higher LOD of 2.847 μM was observed compared with the 3DPEC, along with greater variability in replicate measurements at the same concentration. Furthermore, peak broadening was noted, which extends the NIM oxidation potential window and may increase susceptibility to interference from other species.

Table 1 summarises the recent and relevant studies on electrochemical NIM detection, revealing that the detection limits offered by our all-printed 3DPEC are comparable to those obtained with more sophisticated electrodes. Notably, most such studies are reported only in buffer solutions to avoid fouling or corrosion and to increase the reproducibility, linearity range, and LODs, while our studies were performed in a more complex sample matrix. This summary shows that, despite having a higher LOD compared to some literature reports, the proposed 3DPEC platform can be successfully used to detect NIM in real wastewater samples.

**Table 1:** NIM limits of detection with electrochemical techniques using different electrodes. The term ‘modified’ suggests further electrode modifications, typically by conductive polymers or by grafting receptors that make the sensor disposable. Other acronyms: *CPE*, carbon paste electrode; *GC*, glassy carbon; *CNT*, carbon nanotubes; *rGO*, reduced graphene; *NP*, nanoparticles; *TiO*_*2*_, titanium dioxide; *NS*, nanosilica; *ER-GONRs*, reduced graphene oxide nanoribbons; *MWCNTs*, multi-walled carbon nanotubes; *SPC*, screen-printed carbon; *CD-GrOPUE*, graphite oxide polyurethane modified with β-cyclodextrin; *DHP*, dihexadecylphosphate and acrylonitrile butadiene styrene; *SWV*, square-wave voltammetry; *DPV*, differential pulse voltammetry: *CA*, amperometry

Electrode	Electrolyte	Methods	Linear range (M)	LOD (nM)	LOQ (nM)	Ref
rGO/PEDOT/GC	PBS	SWV	8.0 × 10^−8^–1.9 × 10^−6^	2.4	-	[70]
CB-Nafion/GC	Acetate buffer (pH 4.6)	DPV	2.5 × 10^−7^–1.75 × 10^−6^	60	180	[69]
Cysteic acid/CNTs/GC	0.05 M H_2_SO_4_	DPV	1.0 × 10^−7^–1.0 × 10^−5^	50	-	[71]
CB–DHP/GC	PBS	SWV	3.5 × 10^−7^–2 × 10^−6^	16	-	[72]
ER-GONRs/SPC	PBS	CA	1.0 × 10^−8^–1.50 × 10^−3^	3.5	-	[73]
NS/CPE	PBS	DPV	3 × 10^−5^–1 × 10^−6^	1.4	4.81	[68]
CD-GrOPUE	river	SWV	6.2 × 10^−7^–7.3 × 10^−6^	83	-	[74]
3Ds-NPGE	Urine, river	SWV	10 × 10^−6^–50 × 10^−6^	18	54	[75]
TiO_2_/GC	Acid solutions, urine	DPV	4.0 × 10^−5^–1.0 × 10^−4^	3.4	11.2	[43]
Modified PdNP/GC	Urine, river	DPV	1.3 × 10^−7^–6.0 × 10^−5^	39	132	[45]
MWCNTs/GC	PBS	DPV	3.2 × 10^−7^–6.5 × 10^−5^	160	-	[76]
3DPEC	1:10 (v/v) (0.01 M PBS: sewage)	DPV	1.0 × 10^−6^–75 × 10^−6^	194	649	this work

Spike-recovery experiments were carried out to evaluate the accuracy of the proposed approach to reduce matrix effects. NIM concentrations in sewage samples were determined before (black line) and after analyte addition (red lines) (Fig. 5f). Recovery tests using the standard addition method for 2.0 µM NIM (dark red line) and successive additions of 2.0 µM NIM (light red lines) were successfully performed on sewage water samples prepared with a 1:10 (v/v) dilute mixture (0.01 M PBS, pH 7.01: sewage). The results showed good accuracy, with a recovery rate of 89.6 ± 5.2% (Fig. 5g), confirming the method’s reliability and suitability for wastewater quality control, even in complex sample matrices.

Studies were also conducted to assess the influence of potential interferents commonly found in water and sewage contaminants. Interferents such as paracetamol (PAR), caffeine (CAF), resorcinol (RES), ciprofloxacin (CIP), diclofenac (DIC), and ascorbic acid (AA) were evaluated, and each measurement was repeated three times in the presence of each interferent. SI file, Fig. S13 shows the voltammograms recorded in 0.01 M PBS, pH 7.01: sewage water, in the presence of nimesulide (NIM), followed by the addition of investigated potential interferents in a 1:1 ratio. Oxidation peaks were observed only in the presence of RES and DIC, both of which appeared around 0.58 V, distinct from the NIM peak. This is likely because the other species exhibited oxidation peaks outside the potential window studied and/or had a low electrochemical response under the analysed conditions, resulting in the absence of detectable peaks. The relative percentage change in the electrochemical response of NIM after the addition of potential interfering species is presented in Fig. 5h. Considering a ± 10% variation in the NIM response after the addition of potential interfering species, only the addition of DIC resulted in a slightly higher change, with an 11% decrease in peak current observed across three consecutive measurements. This reduction is likely due to DIC competing for active sites on the electrode surface, thereby accelerating electrode fouling and diminishing the electrochemical response of NIM. Importantly, in all cases, the detection and quantification of NIM remained fully achievable.

**Fig. 6:**
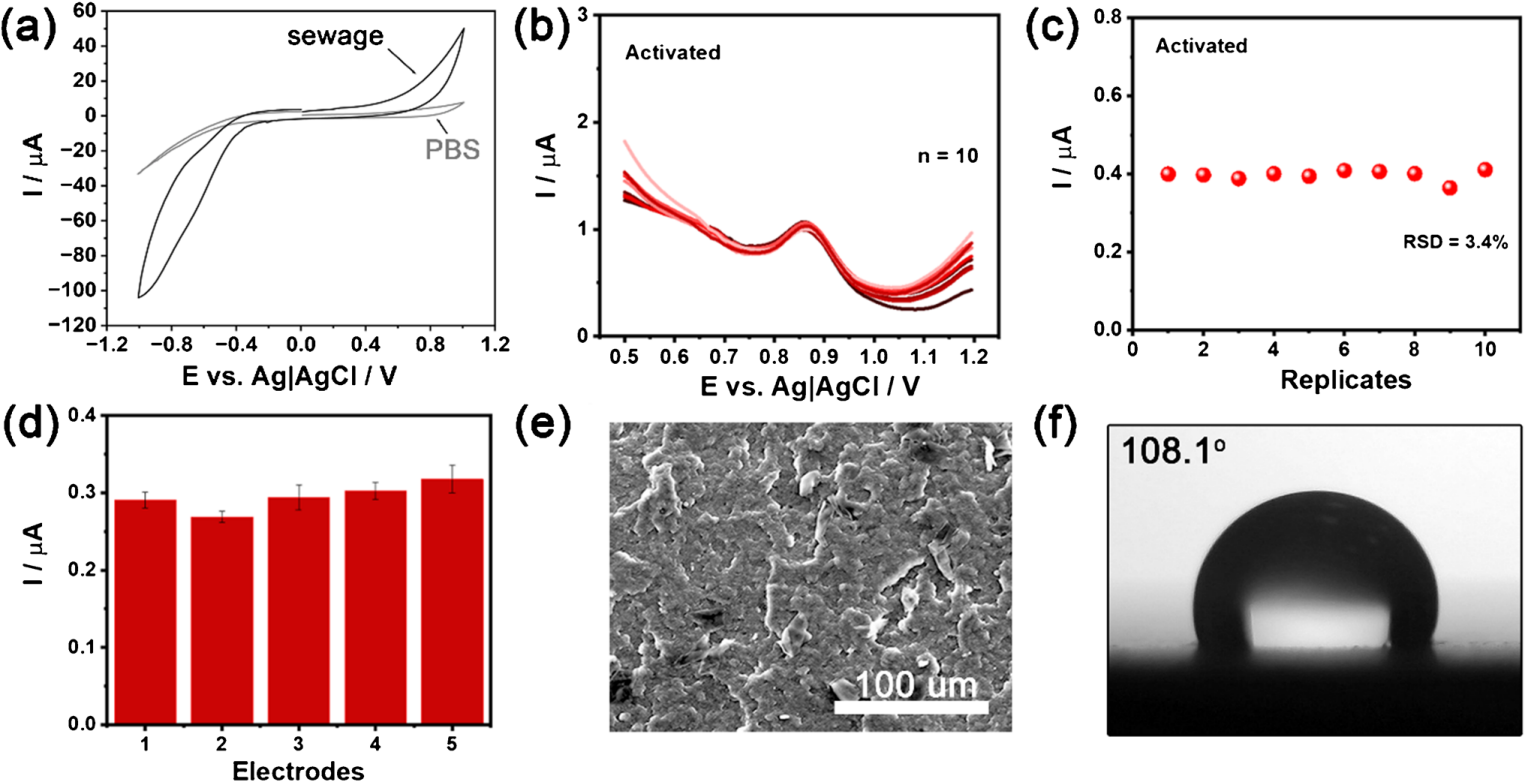
**a** CV scan in the PBS and the industrial sewage in NIM absence, scan rate 100 mV/s; the scan was performed from 0.0 to +1.0 V; +1.0 to −1.0 V; −1.0 to 0.0 V; **b** DPV measurements from ten successive scans (*n* = 10) in the presence of 10.0 µM NIM using the activated 3DP electrode; **c** corresponding variation in current intensities; **d** variation in current intensities from three successive DPV measurements on five different activated electrodes in the presence of 10.0 µM NIM; **e** SEM revealing WE topography and **f** contact angle measurements after ten repetitive NIM analyses. The error bars in **d** represent the standard deviation calculated from three measurements for each electrode

Sewage constitutes a complex matrix that may introduce interfering redox processes, generating overlapping signals that obscure the detection of target analytes. Moreover, NIM oxidation is well-known for introducing fouling effects [77]. Adsorption effects may limit the available ESA and reduce signal repeatability/stability due to changes in the sensor’s electric double layer. The matrix effect on the 3DPEC electrolytic window is presented in Fig. 6a. Its value for the industrial sewage (approx. 1.4 V) is lower compared to the analogous experiment in the PBS (~ 1.9 V); nevertheless, only minor changes in the background currents between both supporting electrolytes were recognised at low and moderate overpotentials.

The interelectrode stability of the sensor was evaluated by 10 consecutive DPV scans with a high NIM concentration of 10.0 µM (Fig. 6b), resulting in an RSD of 3.4% (Fig. 6c), indicating good repeatability. The interelectrode precision, evaluated with five different electrodes at the same concentration (SI file, Fig. S11d), presented an RSD of 6.0% (Fig. 6d), confirming the reproducibility of the activated sensors. Thus, the RSD values for both the repeatability/stability and reproducibility studies of the activated electrode are considered satisfactory (< 10%) for analytical applications [78–80]. The NIM fouling of the 3DP electrode surface after ten repetitive analyses is evident, as shown in the SEM micrograph in Fig. 6e. Additionally, the contact angle of the 3DP electrode increases to 108.1°, confirming the formation of a hydrophobic film that fouls the electrode surface. This film is likely composed of NIM oxidative products and other electropolymerised compounds, such as phenols or quinones [81], which could plausibly be present in sewage. However, as revealed by the electrochemical studies, the changes in surface chemistry after repetitive use of the 3DPEC have a negligible effect on its performance.

Regarding the stability of the polymer matrix in the printed electrodes, although PLA is biodegradable, its aging and mechanical degradation occur slowly, providing a good balance between sustainability and stability [82, 83]. The electrode system remained leak-free after 24 h of exposure to the electrolyte; however, the layered structure inherent in 3D printing can lead to leaks and degradation of PLA over time. Therefore, the use of freshly printed devices is recommended after this period.

## Conclusions

In this work, we proposed and thoroughly characterised an all-3D-printed electrochemical cell (3DPEC). We considered the weaknesses and strengths of the 3DP electrodes used in the electrochemical detection of contaminants in real media and under mechanical stresses, which might affect the operating conditions.

The mechano-electric properties and the micro-computed tomography allowed us to conclude that the introduced tensile stresses will have a negligible influence on the electric and electrochemical properties of 3DPECs. Conductive paths were effectively broken only at *ε* > 0.07 and in the case of very thin, 1-mm conductive CB-PLA stripes. Moreover, a stable interface was obtained via 3D printing between the conductive CB-PLA and non-conductive PLA, which does not spontaneously crack under even considerate forces, while the obtained ESA is reproducible.

The proposed 3DPEC activation by electrochemical saponification in 1 M NaOH under CV cycling effectively increased the ESA, which is nearly equal to the geometric surface area of the WE within 3DPEC. It was also testified that the proposed activation protocol modifies both WE and CE simultaneously. Activated 3DPEC was found to be very effective in detecting NIM in complex matrices, such as industrial sewage, after its 1:10 dilution (v/v). The activation was proven to enhance NIM detection and lower RSD during peak current analysis from 56.4% to 6.8%. The 3DPCE allowed for the determination of a LOD of 0.194 μM in a complex analytical matrix. The recovery rate of 89.6 ± 5.2% confirmed the reliability and suitability of 3DPEC for wastewater quality control, even in complex sample matrices and in the presence of interferents.

By combining mechano-electric durability studies with electrochemical validation, this work demonstrates a versatile, low-cost 3D-printed detection platform that can be adapted to diverse contaminants, providing a sustainable route for practical on-site wastewater monitoring.

## Supplementary information

Below is the link to the electronic supplementary material.

ESM 1 (DOCX 3.72 MB)

ESM 2 (MP4 30.5 MB)

## Data Availability

The data that support the findings of this study are available in an open repository ([https://mostwiedzy.pl/pl/open-research-data/mechanical-durability-and-electroanalytical-performance-of-3d-printed-multi-material-sensors,3250730003020-0](https://mostwiedzy.pl/pl/open-research-data/mechanical-durability-and-electroanalytical-performance-of-3d-printed-multi-material-sensors,3250730003020-0) and [https://mostwiedzy.pl/pl/open-research-data/nimesulide-analyses-with-3d-printed-multi-material-sensors,50108440858356-0](https://mostwiedzy.pl/pl/open-research-data/nimesulide-analyses-with-3d-printed-multi-material-sensors,50108440858356-0)). The 3DPEC used in this study is also available for free in an open repository ([https://mostwiedzy.pl/pl/open-research-data/stl-files-of-all-3d-printable-electrochemical-cell,725074954754294-0](https://mostwiedzy.pl/pl/open-research-data/stl-files-of-all-3d-printable-electrochemical-cell,725074954754294-0)). All data are available from the corresponding author upon reasonable request.
