# Supplementary material for: Multi-material integrated 3D-printed electrochemical detection platform for rapid on-site screening of nimesulide in industrial sewage
Source: Mikrochim Acta. 2025 Oct 27;192(11):757. doi: 10.1007/s00604-025-07634-8 (PMC12559132; doi:10.1007/s00604-025-07634-8)
Supplement: Supplementary file 1 — (DOCX 3.72 MB) [file 604_2025_7634_MOESM1_ESM.docx]

Supplementary Information file for

**Multi-Material Integrated 3D-Printed Electrochemical Detection Platform for Rapid On-Site Screening of Nimesulide in Industrial Sewage**

*Mateusz Cieślik^1,^*, Magdalena Rucka^2^, Gilvana P. Siqueira^1,3^, Adrian Koterwa^4^, Michał Rycewicz^5^, Rodrigo A.A. Muñoz^3^, Robert Bogdanowicz^5^, Jacek Ryl^1,^**

## ^1^ Division of Electrochemistry and Surface Physical Chemistry, Faculty of Applied Physics and Mathematics, Gdańsk University of Technology, Narutowicza 11/12, Gdańsk, 80-233, Poland

## ^2^ Department of Mechanics of Materials and Structures, Faculty of Civil and Environmental Engineering, Narutowicza 11/12, Gdańsk, 80-233, Poland

## ^3^ Institute of Chemistry, Federal University of Uberlândia, Uberlândia, MG 38408-100, Brazil

## ^4^ Department of Analytical Chemistry, University of Gdańsk, Wita Stwosza 63, 80-308 Gdańsk, Poland

## ^5^ Department of Metrology and Optoelectronics, Gdańsk University of Technology, Narutowicza 11/12, 80-233 Gdańsk, Poland

^*^Corresponding authors: [mateusz.cieslik@ug.edu.pl](mailto:mateusz.cieslik@ug.edu.pl) (M.C), munoz@ufu.br (R.M), [jacek.ryl@pg.edu.pl](mailto:jacek.ryl@pg.edu.pl) (J.R.)

**S1. Sewage composition**

**Table S1** – Characteristics of the industrial sewage used in the study. Where: COD - chemical oxygen demand, TSS - total suspended solids, MLSS - mixed liquor suspended solids, VSS - volatile suspended solids.

|  | Parameter | Value |
| --- | --- | --- |
| Organic matter characteristics | COD [mg/L] | 37.4 |
|  | TSS [mg/L] | 3.2 |
|  | MLSS [mg/L] | 1.6 |
|  | VSS [mg/L] | 1.6 |
| Nitrogen forms | N-NH^4+^ [mg/L] | 0.089 |
|  | N-NO^3-^ [mg/L] | 3.01 |
|  | N-NO^2-^ [mg/L] | 0.051 |
| Other ions | Cl^-^ [mg/L] | 86.64 |
|  | SO_4_^2-^ [mg/L] | 37.89 |
|  | S^2-^ [mg/L] | <0.1 |
| Basic physicochemical parameters | pH | 6.92 |
|  | redox [mV] | 210.7 |
|  | Conductivity [µS/cm] | 1261 |

**S2. Supplementary tensile stress tests**


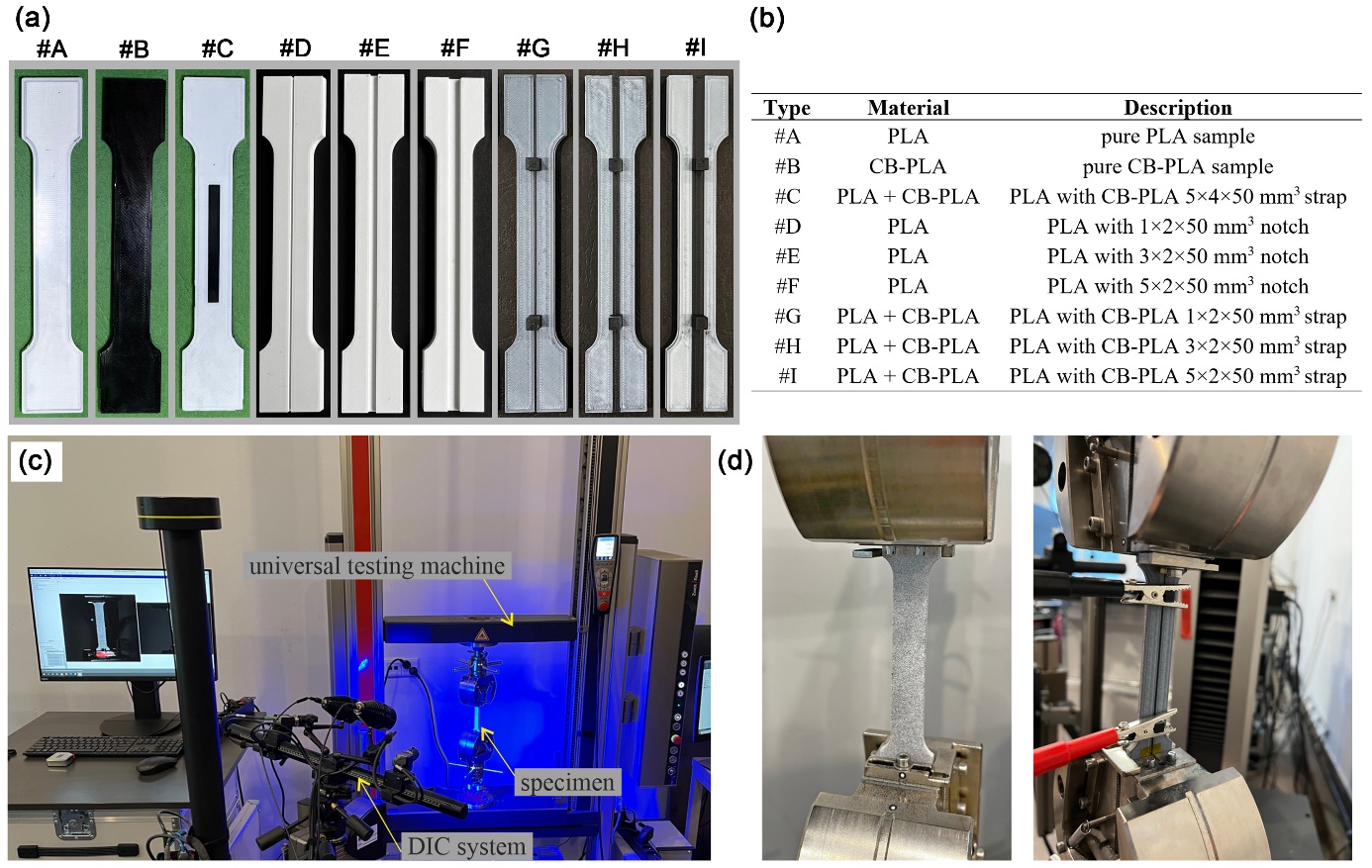

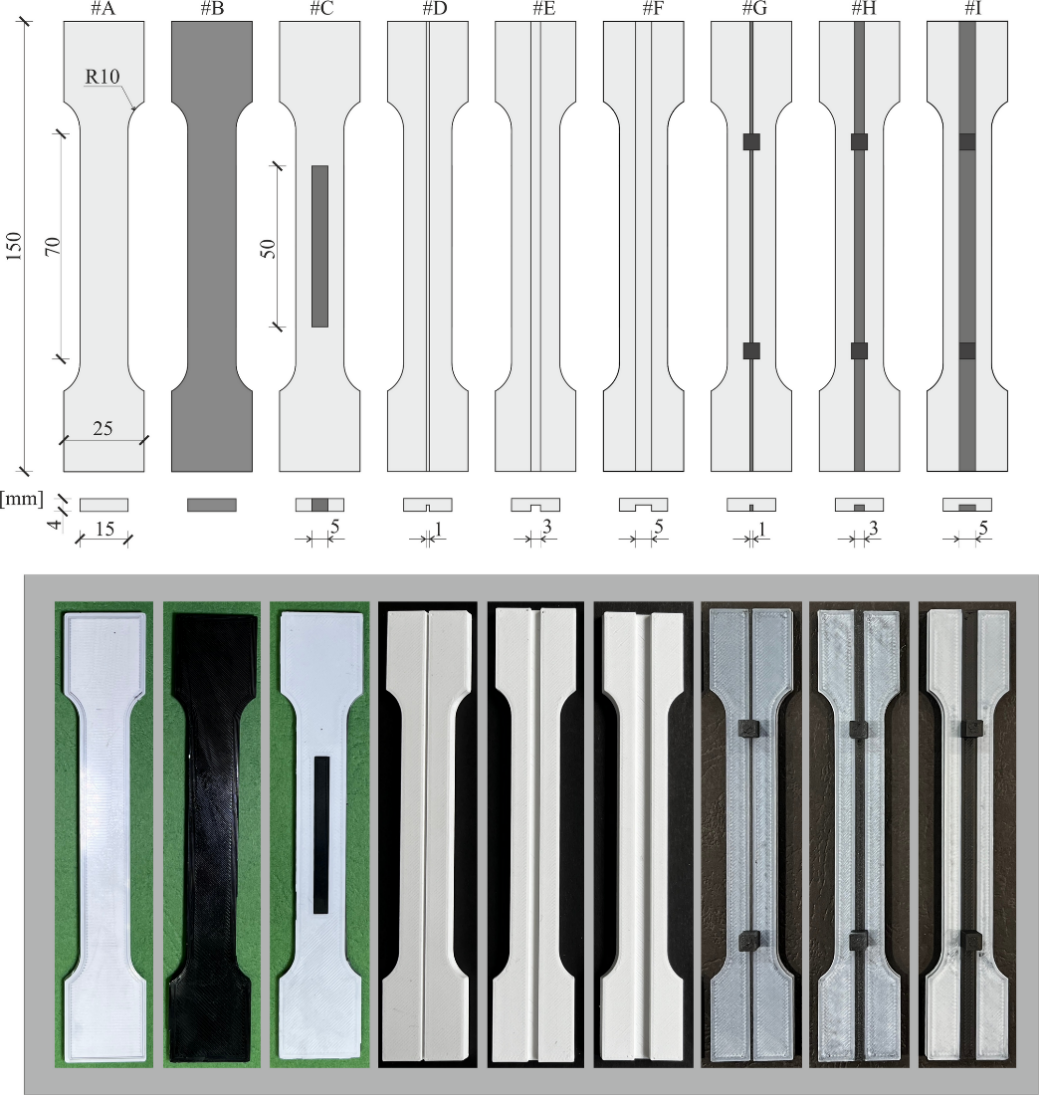


**Figure S1 –** Photos and schemes of samples studied in mechanical and mechano-electric tests: geometry and variants different (#A to #I).


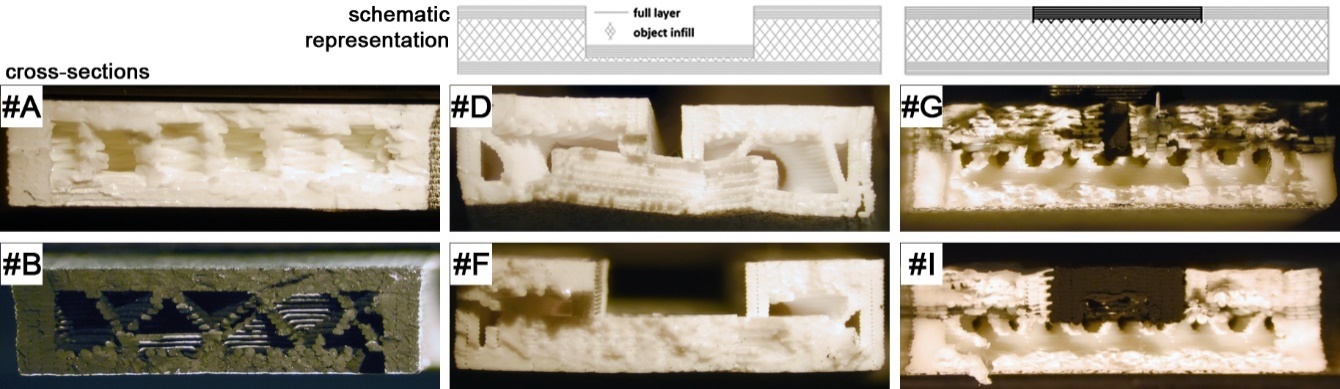


**Figure S2** – The cross-sections of samples used in mechanical and mechano-electric studies, arrangement of layers, and infill.

**Table S2** – Description of the 3DP samples used in the tensile tests


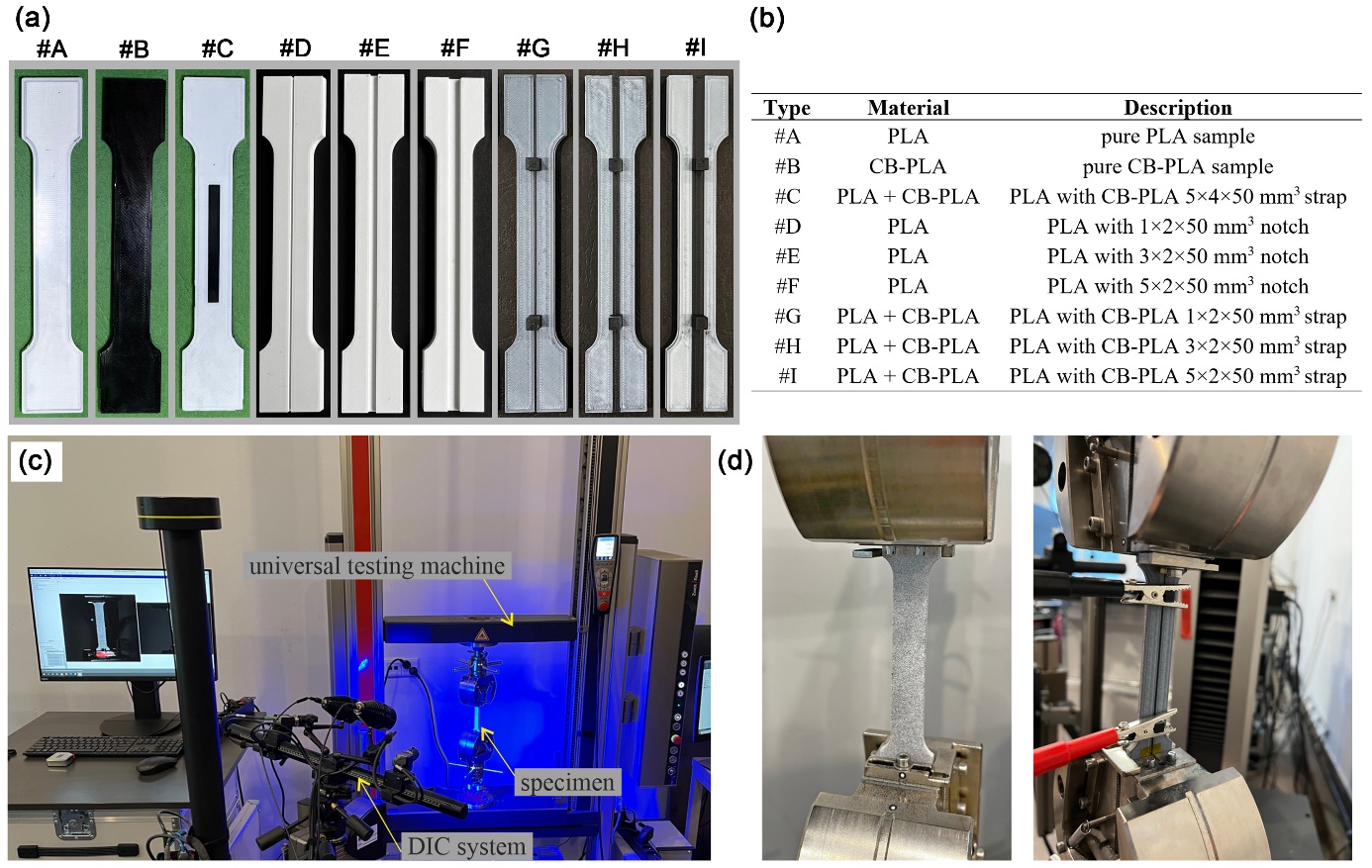


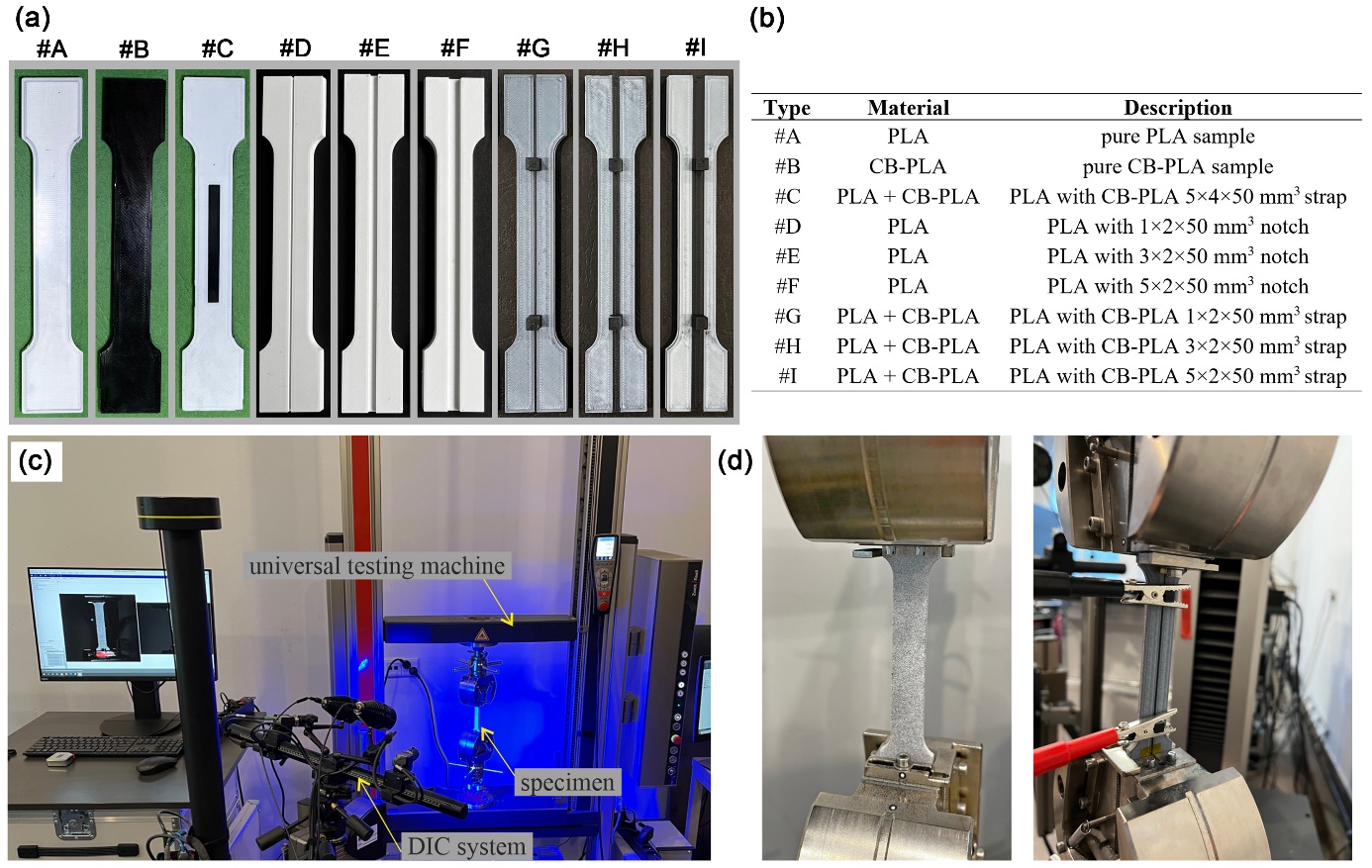


**Figure S3** – The experimental setup for the mechano-electric tests


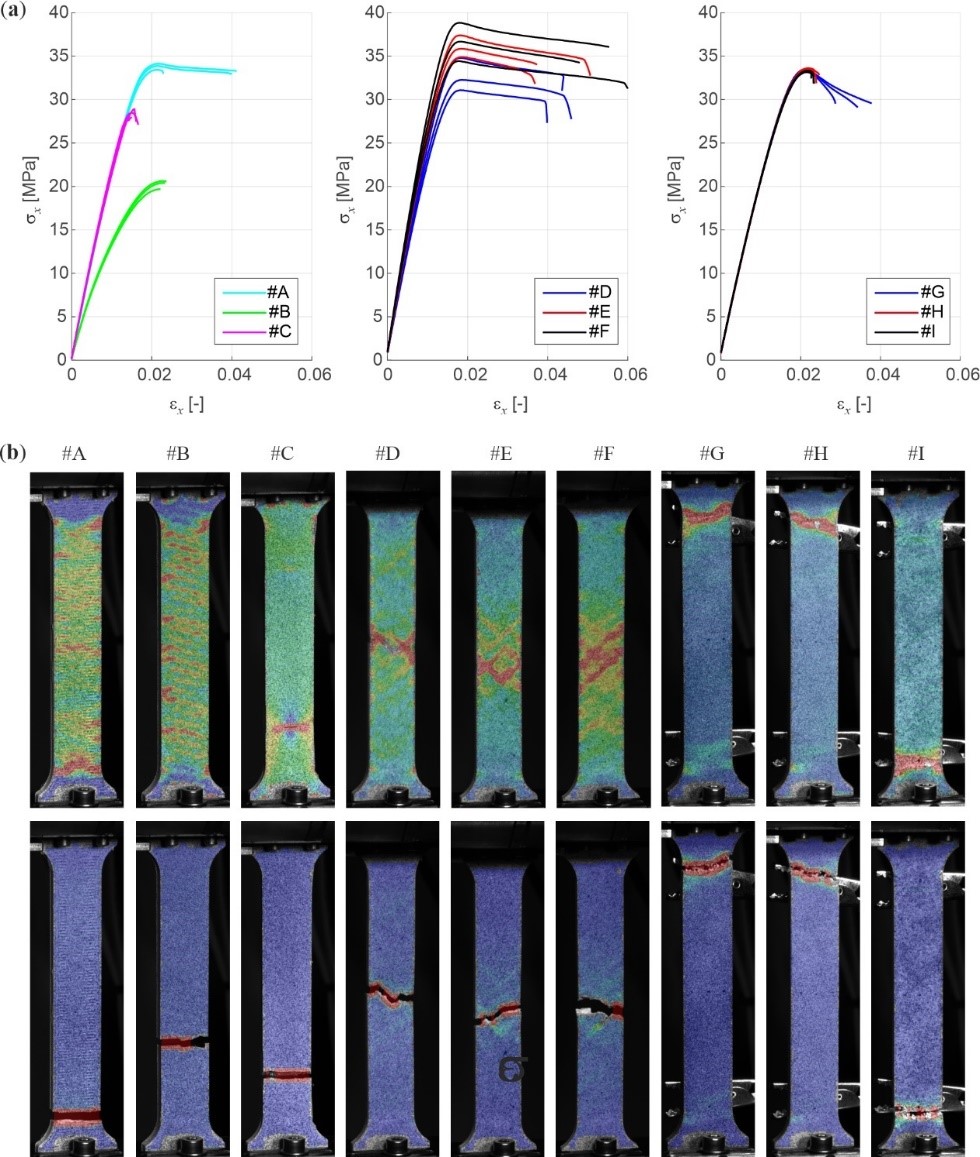


**Figure S4.** a) Stress-strain curves for all specimens tested; b) DIC analysis results in the form of Mises strain maps before failure (first row) and after failure (second row) for all specimens tested #A–#I

**Table S3 –** Tensile strength for all studied samples.

| Material | #A | #B | #C | #D | #E | #F | #G | #H | #I |
| --- | --- | --- | --- | --- | --- | --- | --- | --- | --- |
| Tensile strength [MPa] | 34.133 | 20.400 | 28.486 | 34.792 | 34.883 | 36.664 | 33.482 | 33.462 | 33.329 |
|  | 33.856 | 20.613 | 28.891 | 31.078 | 37.393 | 34.426 | 33.238 | 33.616 | 33.169 |
|  | 33.440 | 19.707 | 27.938 | 32.262 | 35.842 | 38.837 | 33.153 | 33.419 | 33.243 |
| Mean [MPa] | 33.810 | 20.240 | 28.438 | 32.711 | 36.039 | 36.642 | 33.291 | 33.499 | 33.247 |
| St.dev. [MPa] | 0.349 | 0.473 | 0.479 | 1.897 | 1.266 | 2.205 | 0.171 | 0.104 | 0.080 |

For multi-material samples, the introduction of the internal CB-PLA stripes into the PLA backbone results in a tensile strength reduction, down to 28.44 MPa, which occurs primarily due to stress concentration in the corners of the rectangular area. Examination of samples #D–#F and #G–#I allows us to discuss in more detail to what extent the observed tensile strength reduction originates from the presence of the notches and what the role of the CB-PLA addition is, characterised by lower material resistance.

The character of curves for samples #D, #E, and #F differ considerably, with the prime observation being the repeatably lower results compared to the single-material samples. This effect originates from the scheme of work during the tensile process. With the presence of a longitudinal slot through the sample’s entire length, the approach of the state of uniform tension no longer existed and the effect of the bending was significant, influencing large discrepancies in the tensile strength for particular samples. As the width of the notch increased, there was a greater effect of bending and greater strength was obtained in the tensile test. Moreover, the studies performed for samples #G, #H, and #I prove that these bending strengths are solely responsible for the deterioration of the mechanical properties. Here, filling the notch with conductive CB-PLA led to reproducible and similar tensile strength values – 33.291 MPa (#G), 33.499 MPa (#H), and 33.247 MPa (#I) – regardless of the width of the conductive material infill.

The distribution of Mises strains for the samples just before and after failure is shown in **Fig. S4**. The observed failure patterns are related to the structure of the 3DP samples. Single-material samples made of PLA and CB-PLA (#A, #B) broke perpendicularly to the direction of loading. The damage was sudden and occurred after a short phase of localised strain. In sample #C, the horizontal zone of stress concentration occurred at the boundary of the two materials and failure occurred in this area. A different behaviour can be observed for specimens #D, #E, and #F, which did not meet the assumption of uniaxial tension due to the lack of symmetry in one direction. As a result, the samples were subjected to bending during the tensile test in the UTM. The observed stress before failure was localised at an angle of approximately 45°, which means that the influence of shear stresses was significant.

**S3. Electric resistance change upon tensile load**

The resistivity (*ρ*) was calculated according to eq. (S1):

$\rho=R\cdot\frac{S}{l}$ (S1)

where *R* is resistance (Ω), *S* is the cross-sectional area of the conductive path (m^2^), and *l* is the distance between the electrodes (m). Then, the fractional change in resistance (FRC) was calculated following eq. (S2):

$FRC=\frac{\Delta R}{R}$ (S2)

where ΔR denotes the change in resistance, and R_0_ is the initial resistance. The electrode sensitivity was determined based on the gauge factor (*GF*), eq. (S3):

$GF = \frac{FRC}{\epsilon}$ (S3)

where *ΔR* is the change in resistance, *R* is the initial resistance, and *ε* is the applied strain.


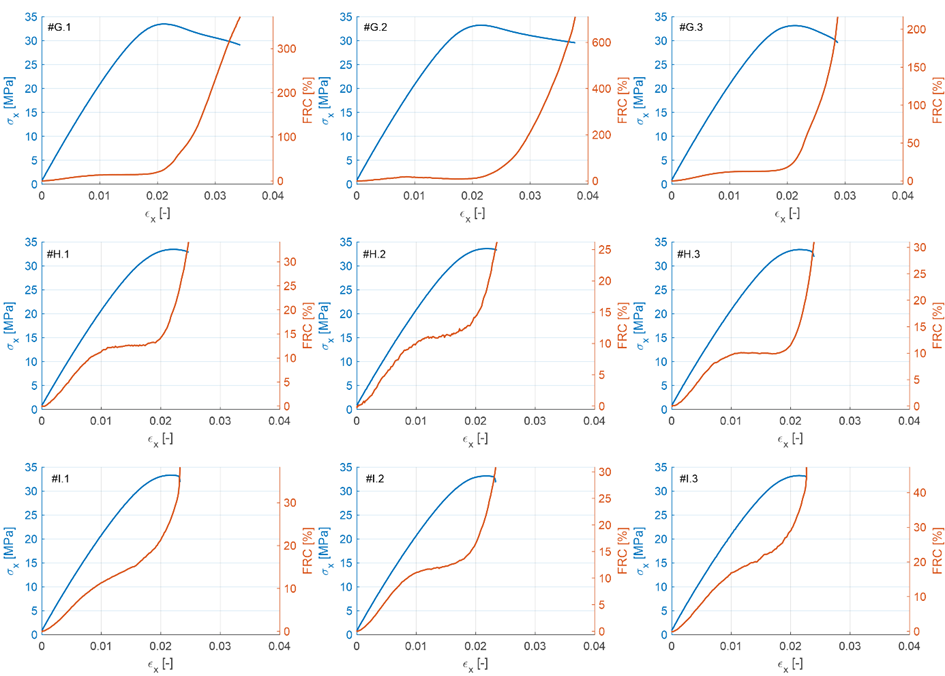


**Figure S5** – Stress-strain curves and FRC-strain curves for all the specimens with conductive stripes and electrodes (#G, #H, #I).


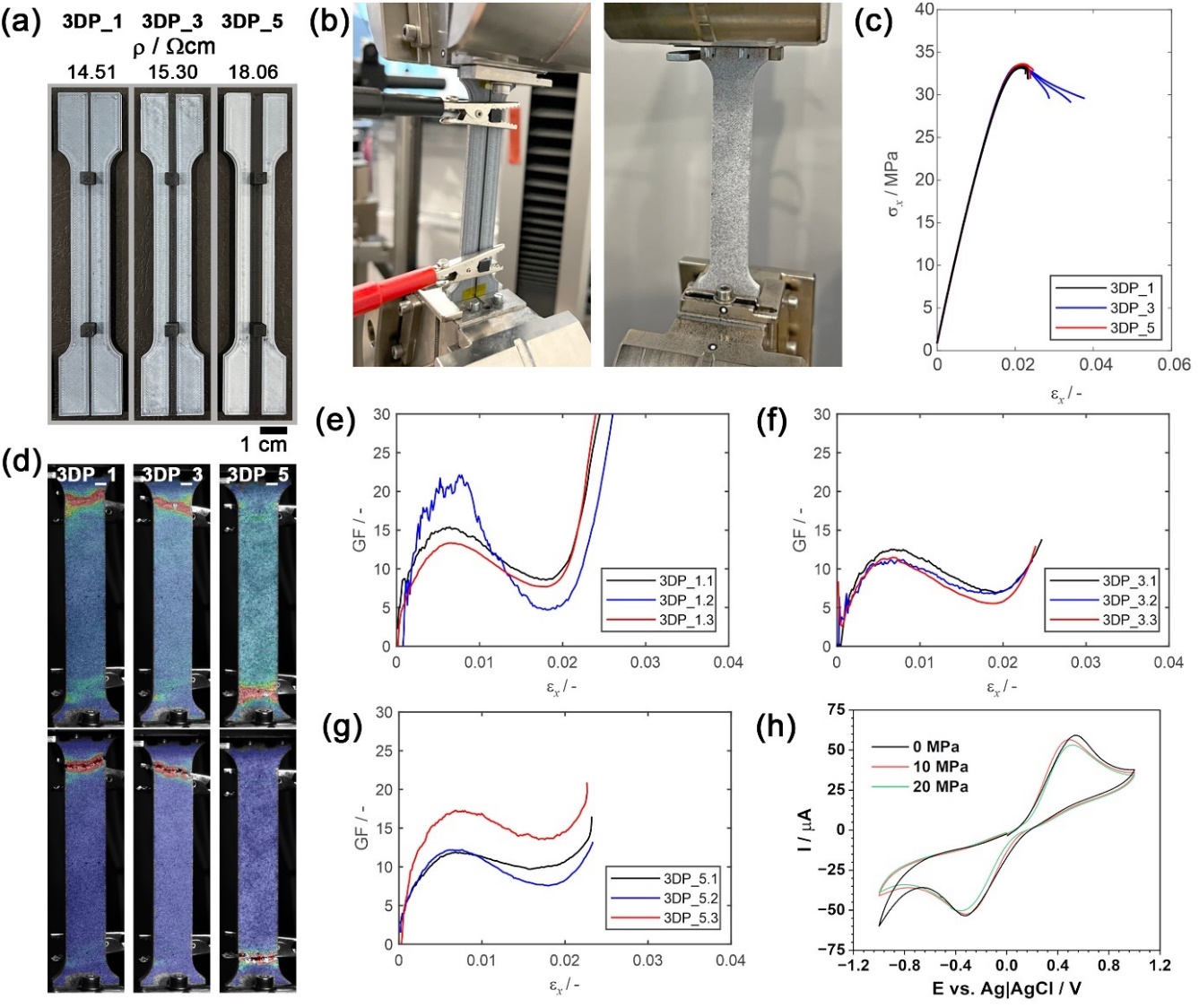

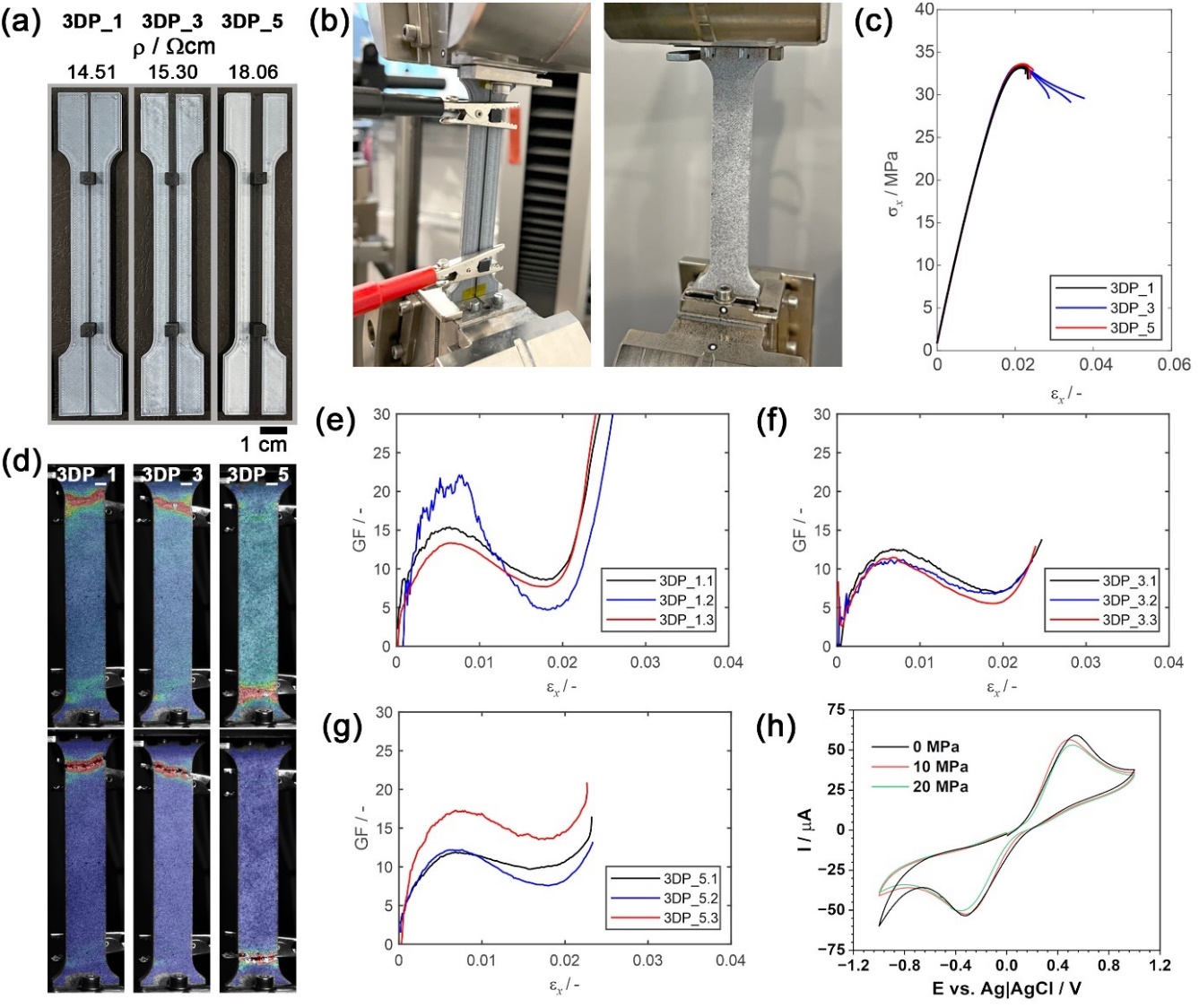


**Figure S6.** (a,b) registered GF as a function of applied strain ε: (a) 3DP_3; (b) 3DP_5.

Notably, the measurement itself may decrease the resistance value due to the temperature change under continuous current flow, where the samples with the smallest cross-sectional area were affected the most, where R decreased by 3% within 5 minutes.


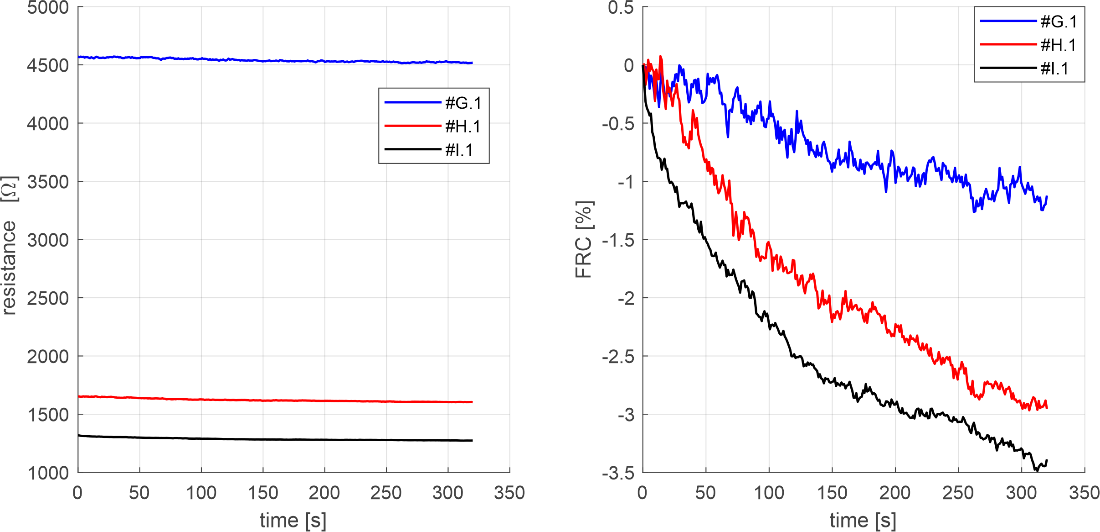


**Figure S7** – Electric resistance of the unloaded samples with different widths of conductive stripes (#G, #H, #I).

**S4. Supplementary electrochemical studies with 3DPEC**

The activation procedure was performed immediately after the electrode was printed. The electrochemical activation was performed in an alkaline medium (1 M NaOH solution, pH = 14) using potentiodynamic polarization from -1.4 to 1.2 V vs Ag|AgCl at a scan rate of 50 mV/s. Each electrode was subjected to a total of 10 polarization cycles.

***
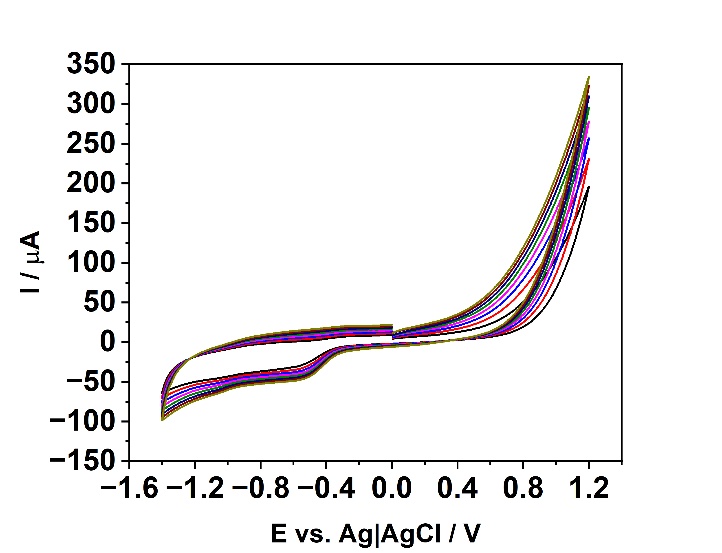
***

**Fig S8 -** CV curves under electrochemical activation in 1 M NaOH solution, potential range from -1.4V to +1.2V. The scan was performed from 0.0 to +1.2 V; +1.2 to −1.4 V; −1.4 V to 0.0 V.


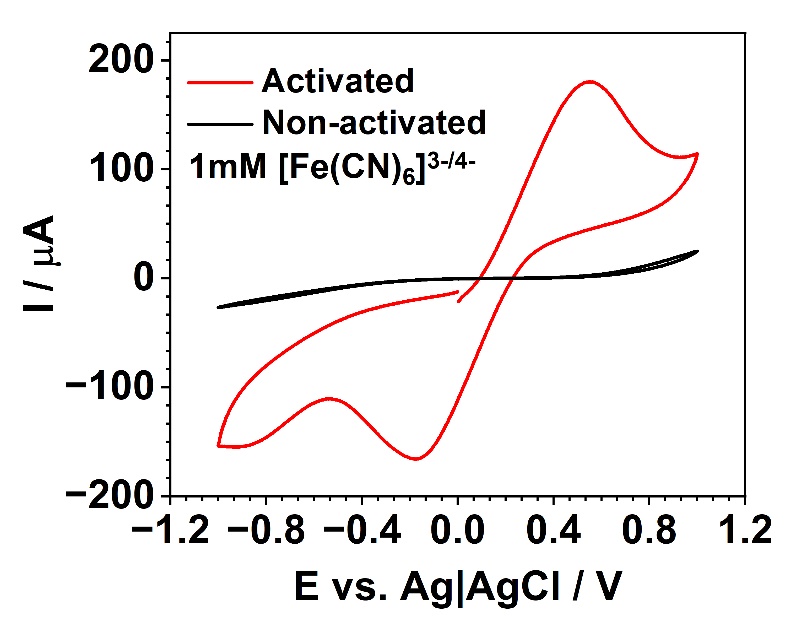


**Figure S9** – CV scan before and after activation of the 3DP electrodes within 3DPEC. Scan rate 100 mV/s. The scan was done from 0.0 to +1.0 V; +1.0 to −1.0 V; −1.0 V to 0.0 V.

**Table S4 –** Results of EIS data decomposition using R(QR) or R(Q(RW) electric equivalent circuit for non-activate and activated 3DP, respectively.

|  | **R / kΩ** | **Q / mFs^α-1^** | **n / -** | **Rct / kΩ** |  |
| --- | --- | --- | --- | --- | --- |
| **Non-activated** | 1.12 | 0.11 | 0.98 | 722 |  |
|  | **R / kΩ** | **Q / mFs^α-1^** | **n / -** | **Rct / kΩ** | **W / Ss^1/2^** |
| **Activated** | 1.14 | 71.85 | 0.83 | 0.35 | 3.66 |


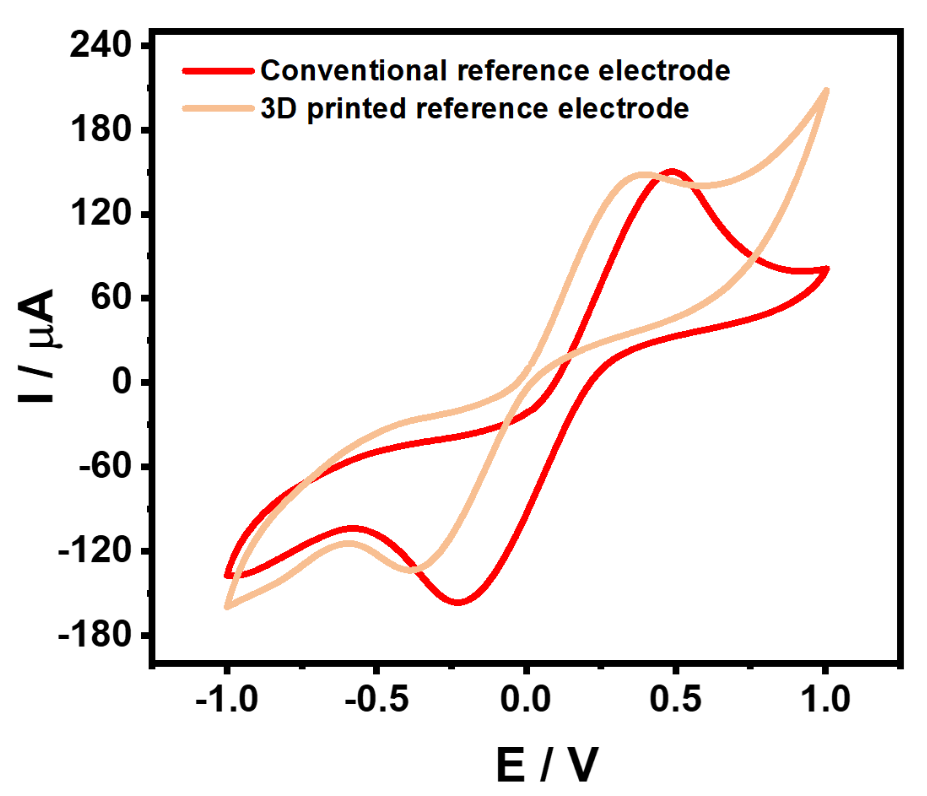


**Figure S10** **–** CV scans in 1.0 mM [Fe(CN)_6_]^4−/3−^ + 0.01 M PBS, revealing the effect of using 3DP pseudo-RE covered with Ag ink on measured redox kinetics, scan rate
100 mV/s. The scan was performed from 0.0 to +1.0 V; +1.0 to −1.0 V; −1.0 V to 0.0 V

**S5. Supplementary electroanalyses in real effluent**


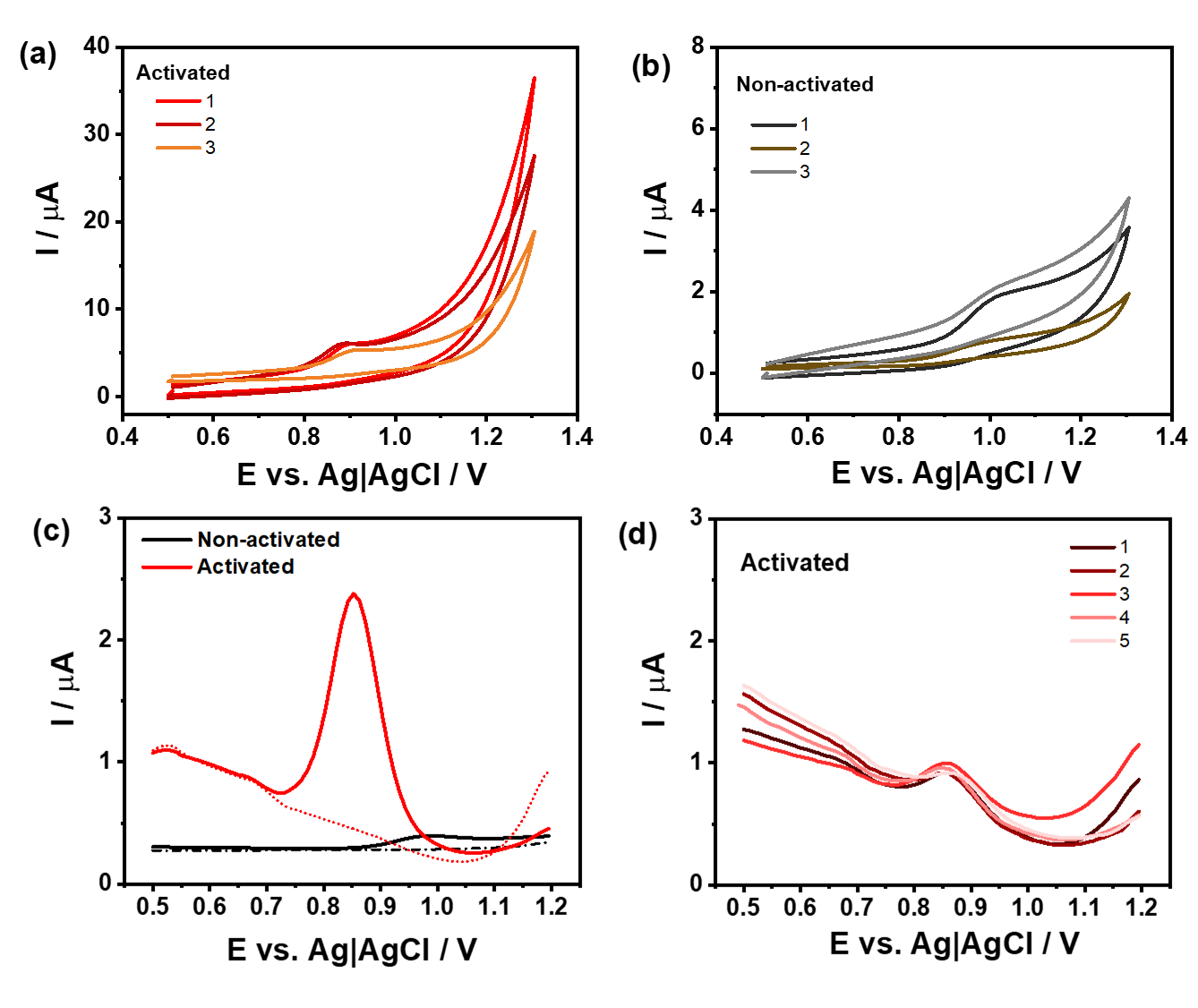


**Figure S11 –** (a) and (b) are CV responses for three activated and three non-activated electrodes, respectively; and the scan was performed from +0.5 to +1.3 V; +1.3 to +0.5 V. (c) DPV analysis of 50.0 µM NIM using 3DP electrodes was performed at activated (red line) and non-activated (black line) electrodes. Nimesulide was dissolved in a diluted mixture of 1:10 (v/v) (0.01 M PBS, pH 7.01: sewage). The dashed lines represent the corresponding blank signals (0.01 M PBS, pH 7.01: sewage). (d) DPV measurements from three successive scans (n = 3) on five different activated electrodes in the presence of 10.0 µM NIM.

The electrochemical detection of NIM using DPV was investigated at both activated and non-activated electrodes, as shown in **Figure S11c**. Blank signal measurements (indicated by the respective dashed lines) in 0.01 M PBS, pH 7.01 : sewage water confirmed the absence of any electrochemical response without NIM. In the presence of 50.0 µM NIM, the DPV anodic current was approximately 35 times higher for the activated electrode, consistent with previous CV results, which showed a 16-fold increase in electrochemical response. The increased value observed in the DPV data, is attributed to the use of the pulsed technique, which minimizes capacitive current and thus amplifies the electrochemical response.


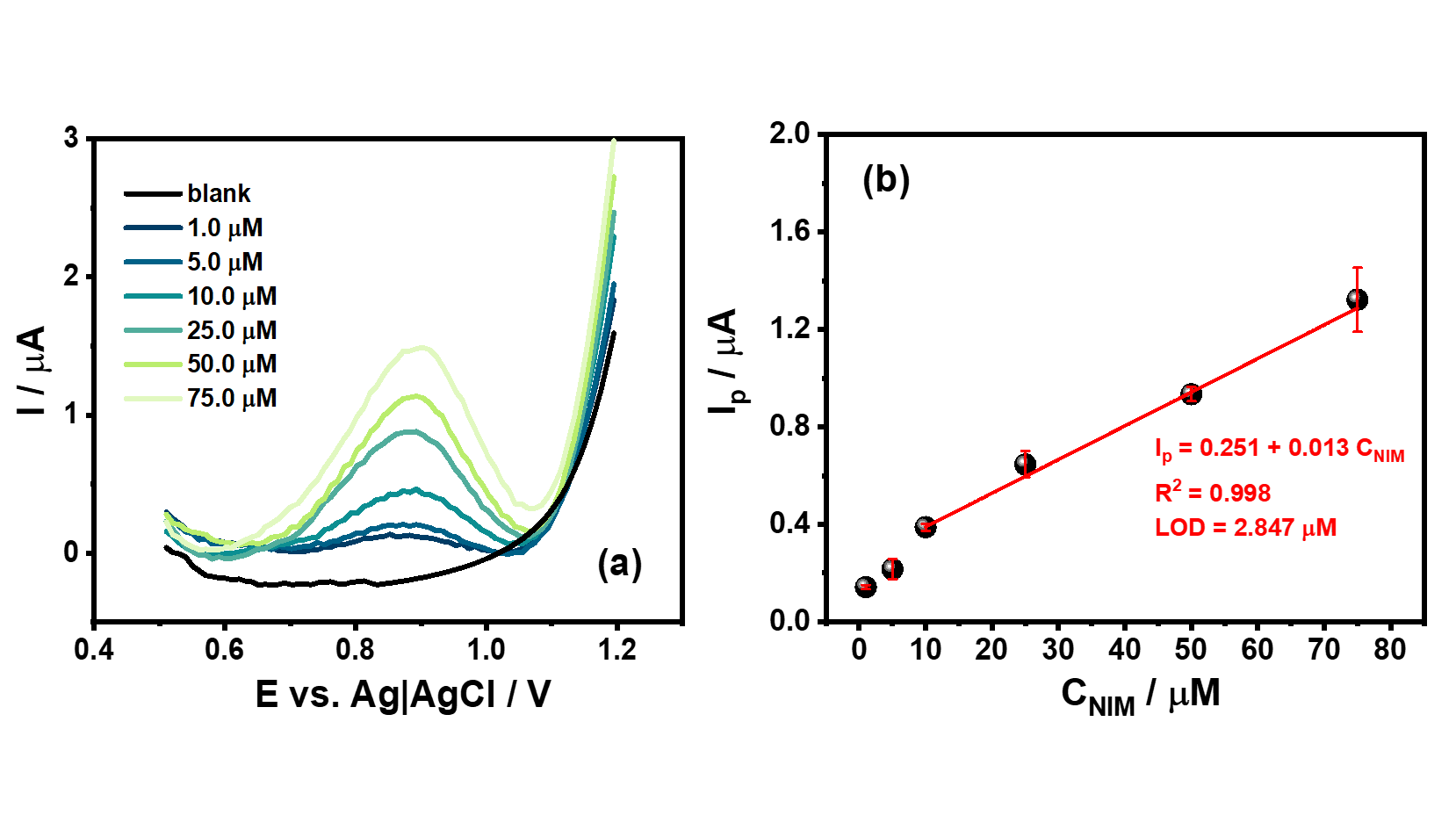


**Figure S12** – (a) DPV analysis with glassy carbon electrode at different NIM concentrations (1.0 to 75.0 μM NIM) in industrial sewage. NIM was dissolved in a diluted mixture of 1:10 (v/v) (0.01 M PBS, pH 7.01 : sewage); (b) Respective calibration curve. The error bars in **Fig. S12b** represent the standard deviation derived from measurements (n=3) at the same concentration.


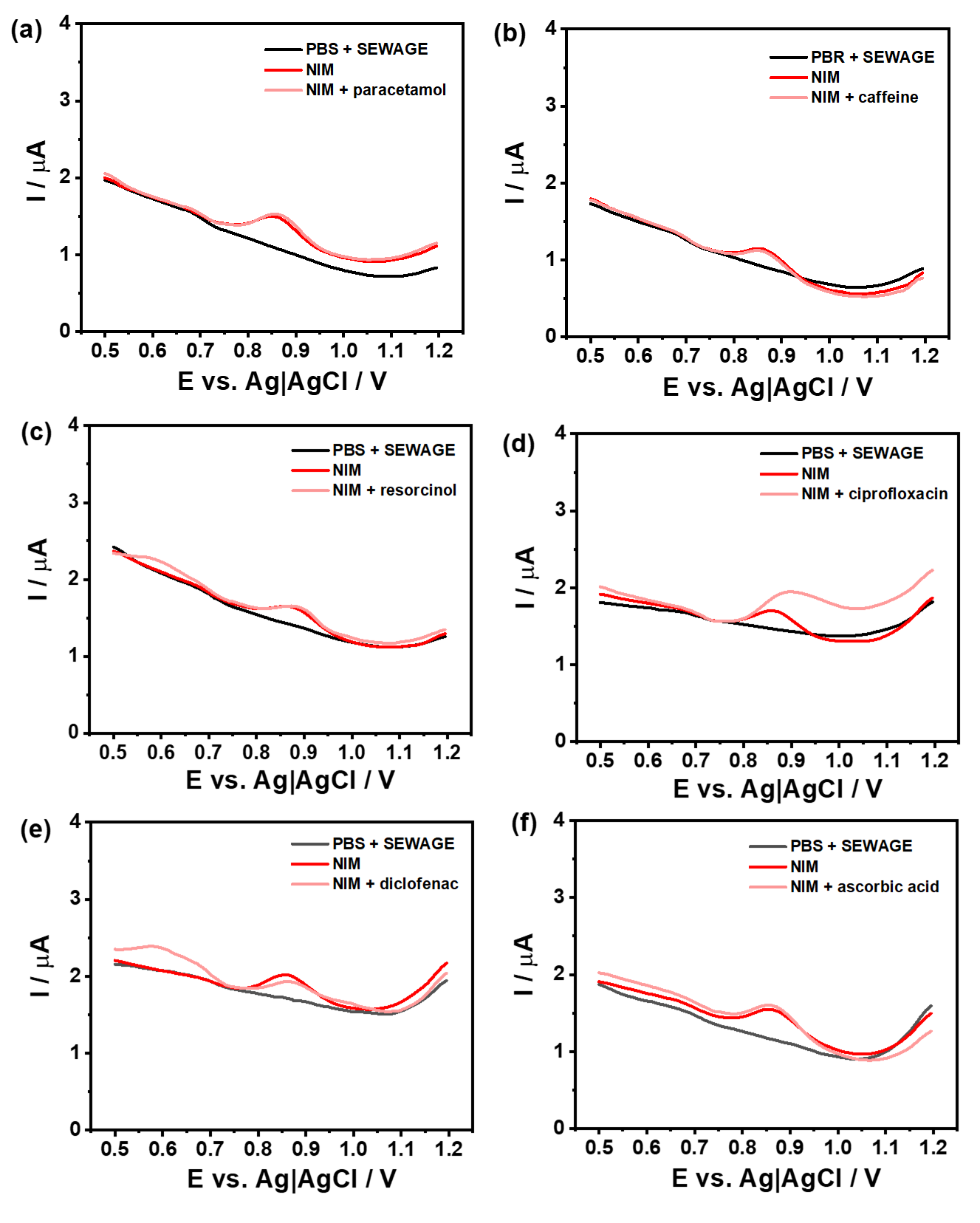


**Figure S13** – DPV measurements performed in the presence of 10.0 µM NIM (dark red lines), and in the presence of 10.0 µM of potential interfering species (light red lines), including (a) paracetamol, (b) caffeine, (c) resorcinol, (d) ciprofloxacin, (e) diclofenac, and (f) ascorbic acid. The black lines represent the corresponding blank signals (0.01 M PBS, pH 7.01: sewage).


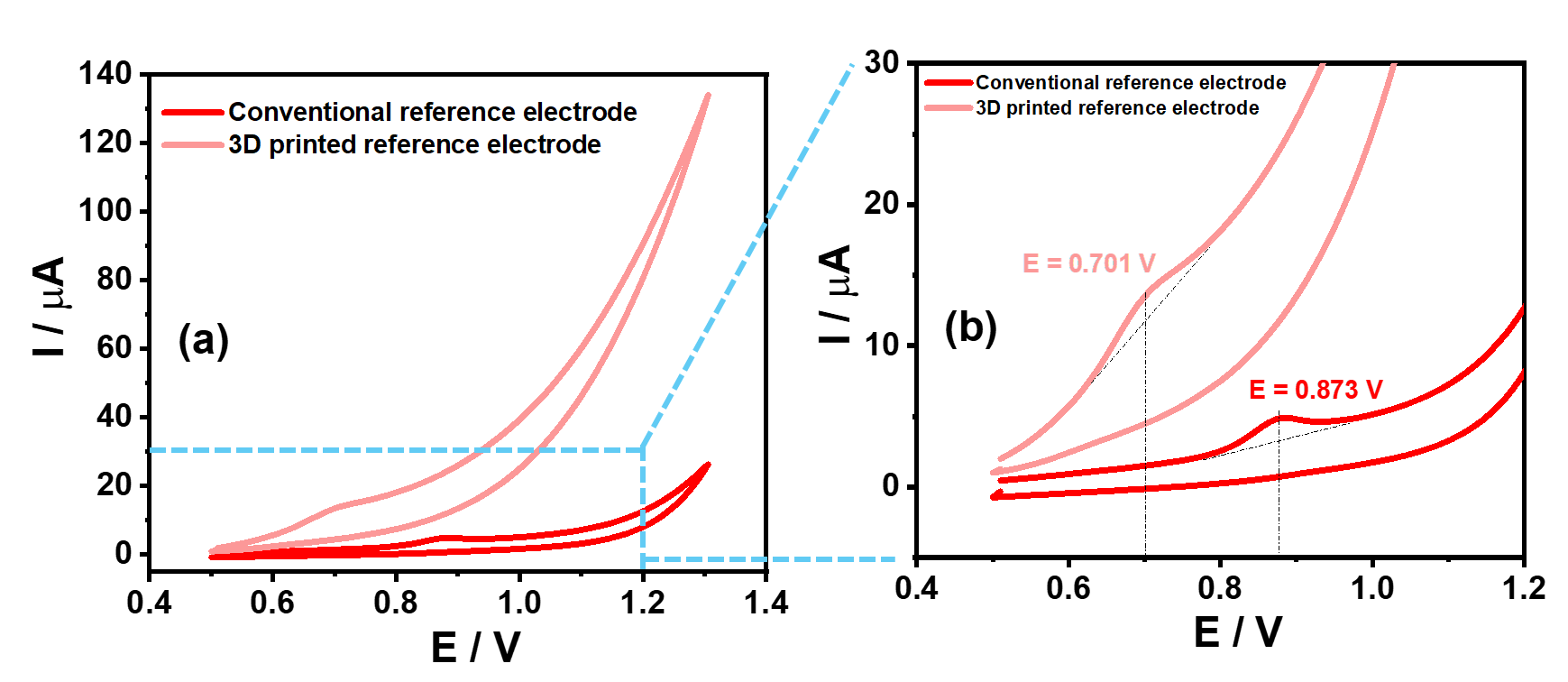


**Figure S14** – (a) CV responses of 0.2 mM NIM using 3DP electrodes using different RE’s (Ag rod and Ag ink-coated 3DPE), with a scan rate of 100 mV/s. Nimesulide was dissolved in a diluted mixture of 1:10 (v/v) (0.01 M PBS, pH 7.01: sewage). The scan was performed from +0.5 to +1.3 V; +1.3 to +0.5 V; (b) same graph, magnified.
